# Natural and Nature-Derived Products Targeting Human Coronaviruses

**DOI:** 10.3390/molecules26020448

**Published:** 2021-01-16

**Authors:** Konstantina Vougogiannopoulou, Angela Corona, Enzo Tramontano, Michael N. Alexis, Alexios-Leandros Skaltsounis

**Affiliations:** 1 Department of Pharmacognosy and Natural Products Chemistry, Faculty of Pharmacy, National and Kapodistrian University of Athens, Panepistimiopolis Zografou, 15771 Athens, Greece; nadia_voug@pharm.uoa.gr; 2 Department of Life and Environmental Sciences, University of Cagliari, Biomedical Section, Laboratory of Molecular Virology, E block, Cittadella Universitaria di Monserrato, SS55409042 Monserrato, Italy; angela.corona@unica.it (A.C.); tramon@unica.it (E.T.); 3 Molecular Endocrinology Team, Inst of Chemical Biology, National Hellenic Research Foundation (NHRF), 48 Vassileos Constantinou Ave., 11635 Athens, Greece; mnalexis@eie.gr

**Keywords:** natural products, phytochemicals, antiviral protease inhibitors, ACE2, coronavirus, SARS-CoV, MERS-CoV, antiviral agents, cytopathic effect, virus-host interactome

## Abstract

The ongoing pandemic of severe acute respiratory syndrome (SARS), caused by the SARS-CoV-2 human coronavirus (HCoV), has brought the international scientific community before a state of emergency that needs to be addressed with intensive research for the discovery of pharmacological agents with antiviral activity. Potential antiviral natural products (NPs) have been discovered from plants of the global biodiversity, including extracts, compounds and categories of compounds with activity against several viruses of the respiratory tract such as HCoVs. However, the scarcity of natural products (NPs) and small-molecules (SMs) used as antiviral agents, especially for HCoVs, is notable. This is a review of 203 publications, which were selected using PubMed/MEDLINE, Web of Science, Scopus, and Google Scholar, evaluates the available literature since the discovery of the first human coronavirus in the 1960s; it summarizes important aspects of structure, function, and therapeutic targeting of HCoVs as well as NPs (19 total plant extracts and 204 isolated or semi-synthesized pure compounds) with anti-HCoV activity targeting viral and non-viral proteins, while focusing on the advances on the discovery of NPs with anti-SARS-CoV-2 activity, and providing a critical perspective.

## 1. Introduction

**Figure 1:**
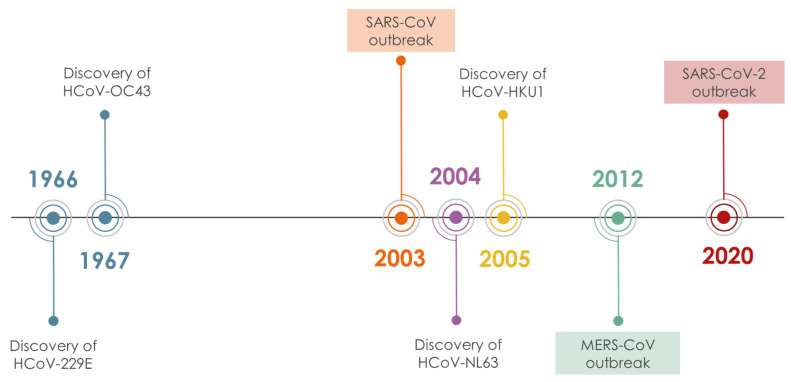
Timeline of HCoV discovery.

Coronaviruses (CoVs), are enveloped, positive strand RNA viruses, with a genome of 27–33 kb, the largest in all RNA viruses. Their virion is spherical (approx. 125 nm diameter), with club-shaped spike-proteins (S protein) that stick out from the surface and result in a crown-like appearance of the enveloped virion [1]. There are seven known human CoVs (HCoVs), (Figure 1) two of which (HCoV-229E, HCoV-NL63) belong to the alpha genera of the subfamily *Orthocoronavirinae* [2] and the remaining five, HCoV-OC43, HCoV-HKU1, MERS-CoV, SARS-CoV and SARS-CoV-2, belong to the beta genera. Most of the circulating HCoVs cause symptoms of common cold, although they occasionally can also cause severe or fatal disease. Three beta-CoVs, namely MERS-CoV, SARS-CoV and SARS-CoV-2, emerged in the last 20 years causing several epidemics of acute respiratory illness associated with high mortality: 10% CFR for SARS CoV-1 and 34% for MERS-CoV [3,4]. The SARS-CoV-2-induced COVID-19 pandemic has caused more than one million deaths since the onset of the disease on 12 December 2019 [5,6]. The genomic sequences of SARS-CoV and SARS-CoV-2 are 79.6% identical and their half-lives in aerosols and in plastic, metal and cardboard surfaces are reportedly similar [5,7]. The comparatively far higher contagiousness and pandemic potential of SARS-CoV-2 are thought to reflect in part the substantial prevalence of undocumented contagious infections compared to the documented ones [7]. The contagiousness of the virus renders its containment difficult and the demand for prophylactic and therapeutic agents an utmost necessity that drives the scientific community in a massive screening effort. In this scenario, bioactive molecules from the vegetable kingdom are a source worthful to mine. The modern tools of NPs chemistry (fast identification, dereplication, fast chemical profiling, in silico screening) and biological evaluation (high throughput in vitro screening assays, live infection assays, high throughput genomics and proteomics of host’s response to infection) provide ample means to explore plant biodiversity for discovery and/or development of NPs/SMs that can help cope with COVID-19 and here we summarize the efforts accomplished up to date.

The aim of this review is to summarize the anti-HCoV activity of natural products and derivatives thereof and their potential for prevention and/or treatment of coronavirus infections, COVID-19 in particular. We have reviewed the bibliography related to human coronaviruses and natural products since the discovery of the first HCoV in the 1960s, up to December 2020. Scopus, PubMed/MEDLINE, Web of Science, and Google Scholar, were employed for the literature search. A total of 135 references related to CoVs and NPs were assessed, while results corresponding to non-human coronaviruses were excluded. Finally, 52 original publications presenting results on anti-HCoV activity were incorporated in the review, corresponding to 19 total plant extracts and 204 isolated or semisynthesized pure compounds. 

## 2. SARS-CoV-2 and SARS-CoV: Structural Aspects and Therapeutic Targeting

SARS-CoV is by far the most studied HCoV among the seven strains. It has a genome size of almost 30 kb [4]. Electron microscopy has shown that the viral particles have an average diameter of 80–140 nm and bear characteristic proteinaceous spikes (S) on the envelope. The surface S protein, encoded by the most variable structural gene of the genome [8], is involved in attachment and entry into the host cell, by interacting with key host cell receptor, the angiotensin-converting enzyme 2 (ACE2) [9], and thus it is the main target for antiviral peptides and antibodies. The ACE2 is a metalloprotease expressed in the lung, intestine, liver, heart, vascular endothelium, testis and kidney cells [4]. Entry into a host cell is an essential step of transmission of SARS-CoV. S protein binds to ACE2 through its S1 subunit but requires at least two protease cleavages to drive fusion through its S2 subunit. Proteolysis at the S1/S2 boundary and a second site within S2 is known to release a fusion peptide, which anchors on the host cell membrane to trigger a change of S2 conformation that promotes virus insertion into the target cell [10]. Several proteases, including extracellular proteases (e.g., elastases in the respiratory tract) and host cell surface proteases (e.g., transmembrane protease serine 2, TMPRSS2) could cleave S protein to render it fusion-competent. TMPRSS2 is reportedly requisite for S protein priming and S2-driven fusion of viral and host membranes [11,12]. However, SARS-CoV can also enter host cells through endocytosis and processing for fusion by endosomal cysteine proteases (e.g., cathepsin L), whose activity is however not essential in presence of TMPRSS2 [13,14]. A combination of a TMPRSS2 inhibitor and a cathepsin L inhibitor can effectively block SARS-CoV entry to host cells [15]. There are two major domains in S1, the N-terminal domain (S1-NTD), which is known to bind sugars, and the C-terminal domain (S1-CTD), which is responsible for recognizing the host receptor. ACE2 interacts specifically with a single region of S1-CTD, known as receptor binding domain (RBD). Host susceptibility to SARS-CoV infection is primarily determined by the affinity of RBD for ACE2 [16]. The RBD binds to the outer surface of the peptidase domain of ACE2, without involving or affecting the peptidase activity, which is not requisite for virus entry [10]. Determination of the crystal structure of SARS-CoV RBD complexed with ACE2 (PDB code: 2AJF) and functional studies have resolved several mechanistic aspects of ACE2 recognition by SARS-CoV and can help to develop effective vaccines. 

SARS-CoV-2 is very closely related to SARS-CoV. The S1 and S2 subunits of SARS-CoV and SARS-CoV-2 are largely conserved, with the S2 subunits sharing the higher sequence identity (88%). Nevertheless, the SARS-CoV-2 S protein contains a furin-cleavage site that is involved in the biogenesis of the virus and differentiates it from all other SARS-like coronaviruses [17,18]. Like SARS-CoV, SARS-CoV-2 binds to ACE2 with high affinity [11,19,20]. The crystal structures of SARS-CoV and SARS-CoV-2 RBD bound to the S-binding domain of ACE2 are nearly identical [21]. The cryo-EM structure of SARS-CoV-2 S protein trimer is also reported [18,22]. The RBD is encoded by the most variable gene of SARS-CoV-2 genome. Although most of the amino acid residues that are essential for binding of SARS-CoV RBD to ACE2 are conserved in SARS-CoV-2 RBD, the latter is not recognized by several monoclonal antibodies directed to SARS-CoV RBD [22]. However, sera from mice immunized with SARS-CoV could inhibit SARS-CoV-2 host cell entry by nearly 90% [18]. Both SARS-CoV and SARS-CoV-2, use TMPRSS2 for S protein priming, and camostat mesylate (an approved TMPRSS2 inhibitor) was reported to partially inhibit SARS-CoV-2 cell entry. However, in cell lines expressing both TMPRSS2 and cathepsins B and L (CathB/L), full inhibition was observed with a combination of camostat mesylate and E-64d, an inhibitor of CathB/L [11].

The genome of both SARS-CoV and SARS-CoV-2 encodes also several non-structural proteins (Nsps), including RNA-dependent RNA polymerase (RdRp, nsp12), [23,24] helicase/NTPase (simply helicase) [25], and two cysteine proteases involved in viral nascent polyprotein processing in different sites, namely 3CL^pro^ (chymotrypsin-like protease, also known as the main protease, M^pro^) [26,27] and PL^pro^ (papain-like protease) [28]. RdRp is the catalytic center of the replication/transcription complex formed by its interaction with multiple Nsps (nsp7, nsp8), [23] while it is the target of remdesivir, a nucleotide analogue that has recently been approved by FDA, for COVID-19 treatment [29,30]. The two cysteine proteases of the two SARS-CoVs, share a great similarity and are an attractive target for antiviral discovery. 3CL^pro^ [31] and PL^pro^ [32] participate in the proteolytic cleavage cascade of the viral polyproteins (1a and 1ab), which is essential for the maturation and replication of the virus. Specifically, 3CL^pro^ cleaves pp1a and pp1b in more than 11 positions, and also releases the key-factors for replication RdRb and helicase [33]. PL^pro^ cleaves the polyprotein in 3 sites and also has a deubiquitinating (DUB) and deISGylating (deISG) activity. This latter property poses implications for the host immune response in viral infection. It has been shown that ISGylation is important for viral clearance and protection, while ubiquitination is implicated in innate immune signaling pathways [34].

## 3. NPs with Anti-HCoV Potential 

**Figure 2:**
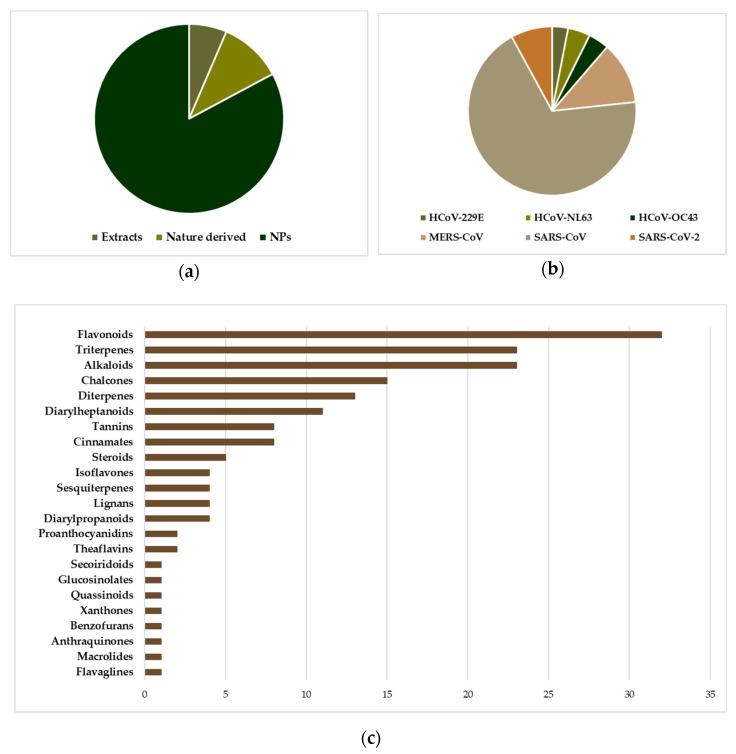
The position of natural products implicated in anti-HCoV research. (**a**) Distribution of entities (extracts, NPs, Nature derived molecules) subjected to anti-HCoV assays (cell based or viral enzyme inhibition), in a total of 326 experiments corresponding to 197 entities. “Nature derived” refers to synthetic/semisynthetic NPs with a basic core inspired by NPs. (**b**) The prevalence of SARS-CoV testing among several NP entities. (**c**) The distribution of the 168 pure NPs tested in various anti-HCoV assays, with respect to the chemical category. Data derived from Tables S1–S4, Supporting information.

Before the emergence of SARS-CoV and MERS-CoV, the investigation of NPs as anti-HCoV agents was limited to only a few studies. The two epidemics have spurred however the interest in the discovery of anti-HCoV agents, including the investigation of agents active against the “common cold” HCoVs, 229E, NL63, OC43 and HKU1. Screening chemical libraries against target proteins and/or viral replication in cell-based assays have been extensively employed, resulting in the discovery of several antiviral natural products/small molecules (NPs/SMs) [35,36,37,38]. It was found that NPs/SMs are a rich source of drug potential leads against coronaviruses due to their pronounced structural diversity and complexity. Several compilations of pre-2020 findings on NPs/SMs with activity against coronaviruses were recently reported, some of which could serve as leads for the development of new drugs [39,40,41,42,43,44,45,46,47,48,49,50]. Among the identified molecules the majority is represented by NPs (Figure 2a) and most of them have been found as inhibitors of SARS-CoV (Figure 2b). Among them, the most numerous classes are flavonoids, triterpenes and alkaloids (Figure 2c), which is not surprising since in these classes reside the greatest number of NPs described as endowed with inhibitory activity against replication of multiple different viruses. In addition, flavonoids are a class of NPs present in almost every plant species and are strongly represented in various NP-based chemical libraries available for screening. 

In the majority of studies, the initial screening involves the addition of the compound or extract to be tested in the normal cell culture, and a subsequent inoculation with the desired viral strain. The cytopathic morphology of the cells is evaluated under a microscope, while the cytotoxicity of the compound/extract is evaluated in normal cells and compared. Compounds with low cytotoxicity are then typically evaluated in multiple concentrations for their ability to inhibit viral replication (EC_50_) and host cell growth (CC_50_), and the selectivity index (SI, CC_50_/EC_50_ ratio) is used to identify the lead compounds, where higher SI refers to compounds more active than toxic. To a first step in investigating the molecular mechanisms underlying the antiviral activity, interactions of the lead compounds with identified targets, such as viral proteases, host proteases, or viral S proteins, are evaluated. Due to the additive complexity of the host/virus system, it is rather difficult to correlate cytopathic effects (CPE) to specific target interaction/inhibition, and this is the case for most natural products tested for their anti-HCoV potential, where extensive mechanistic studies are limited. Available in the Supporting Information are Table S1 (Plant extracts tested for anti HCoV activity in various strains), Table S2 (Pure natural products and nature-derived compounds tested for anti HCoV activity in various strains), Table S3 (Inhibitory activity of extracts against the main protease of SARS-CoV, 3CL^pro^) and S4 (Inhibition of HCoV enzymes from natural and nature-derived products), summarizing the results of anti-HCoV evaluation (viral enzyme inhibition, cell-based assays against viral propagation) of NPs against all strains.

### 3.1. “Common Cold” HCoVs

HCoV-229E (alpha-HCoV) and HCoV-OC43 (beta-HCoV) were identified in the mid-60s, as two of the strains causing the common cold [51,52,53]. The presence of non-typical respiratory viral strains was suspected since 1962, yet difficulties in cultivating viruses impeded scientists from discovering those strains earlier [52]. Van der Hoek, reviews the discovery and clinical manifestations of those “old HCoVs” [54]. Replication of both strains in the human body produces similar symptoms, such as excess nasal excretions, light cough, malaise and rarely, fever. Nevertheless, those strains were proven serologically unrelated, as antibodies isolated from OC43-infected persons, were unable to neutralize 229E. Lower respiratory tract infection was found more common in children and the elderly, manifested with bronchitis, bronchiolitis, croup, and pneumonia, while healthy adults presented mainly common cold. The prevalence of old CoVs infection in the general population has been studied in several cases, although difficult because of the varying clinical manifestations. Reverse-transcription PCR (RT-PCR) has been used for the identification of HCoV viral strains, in one of the largest studies among the UK population, revealing the autumn-winter seasonality of OC43, the lack of seasonality for 229E and its high detection in immunocompromised patients [55]. There are several indications that viral infection from 229E reprograms the host cell transcriptome with a subsequent fine-tuning of NF-κΒ, in order to ensure optimal replication [56].

**Figure 3:**
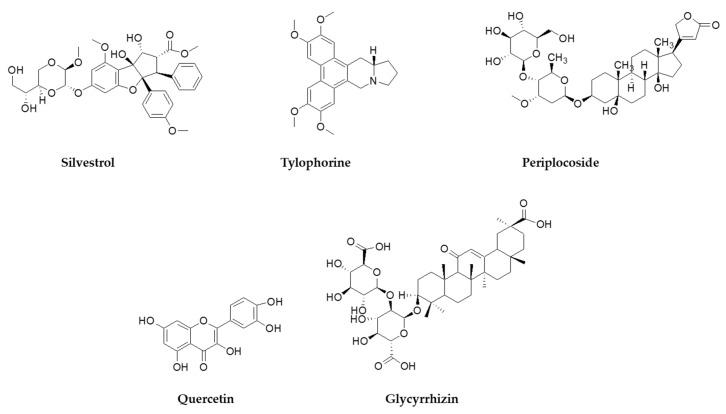
NPs active against HCoVs replication.

HCoV-229E uses human aminopeptidase N/CD13 glycoprotein (APN) to infect host cells. APN is a metalloprotease expressed in lung, intestinal, and kidney epithelium. The receptor was discovered in 2004, nearly 40 years after the discovery of the virus [57], while the X-ray crystal structures of the RBD of the S protein in complex with hAPN, as well as the electron cryomicroscopy structure of the 229E S-protein, was reported in 2019 [58]. Characterization and mapping of the fusion core of the S protein revealed that the domain HR1 folds into an unusually long helix, in post-fusion conformation, while there are very strong interactions between domains HR1 and HR2 that should be taken into account when designing antiviral agents mimicking HR2 binding to HR1 [59,60,61]. As in many CoVs, the S protein of 229E is cleaved by TMPRSS2, human airway trypsin-like protease (HAT) [62], and cathepsin L (CPL) [63]. Recent data show that cleavage into S1/S2 subunits by TMPRSS2 is not a prerequisite for infection, as the virus can use alternatively the endocytic pathway [64]. HCoV-NL63 is closely related to 229E and was discovered in 2004 in infants and immunocompromised patients [65,66]. NL63 uses the same receptor as SARS-CoV for viral entry (ACE2) [67], while the crystal structure of the S protein RBD in complex with ACE2 was reported in 2009, revealing that although NL63 and SARS recognize the same receptor regions, their RBD cores have no structural homology [68]. Currently, there are very few attempts to investigate the antiviral activity of extracts against 229E and NL63. A standardized extract of *Pelargonium sidoides* (Geraniaceae) displayed weak activity against 229E (EC_50_ = 44.50 μg/mL) and is approved in Germany as a phytotherapeutic against respiratory infections, was tested in a panel of respiratory viruses and showed a weak activity against 229E with an EC_50_ of 44.50 μg/mL [69]. In an early study, *Stevia* sp. extracts rich in steviosides used as a natural sweetener, showed virostatic and virucidal activity against 229E, yet there was no follow-up of the results reported [70]. Indole alkaloids indigodole B and tryptanthrine, isolated from *Strobilanthes cusia* (Acanthaceae) have been shown to reduce NL63 titers in early and late stages of LLC-MJ2 cells infection, while inhibiting enzymes vital for viral replication (PLP2, RNA polymerase) [71]. Isolated NPs/SMs that have been tested for their effect in 229E in vitro replication include saikosaponins isolated from *Bupleurum* and *Heteromorpha* species (Apiaceae) [72], pentacyclic triterpenes isolated from *Euphorbia neriifolia* (Euphorbiaceae) [73], xanthones isolated from *Calophyllum blancoi* (Guttiferae), [74] and the flavagline silvestrol that is isolated from plants of the genus *Aglaia* (Meliaceae) [75]. Silvestrol (Figure 3) in particular, had an EC_50_ in the nanomolar range (3.0 nM) in infected MRC-5 cells and an excellent Selectivity Index (>3300), showing no significant cytotoxic effect in the primary cells used [75]. Tacrolimus, an immunosuppressant drug isolated from the soil bacterium *Streptomyces tsukubaiensis* [76], was found to inhibit replication in NL63 and 229E with an EC_50_ of 5.1 and 5.4 μM, respectively [77].

HCoV-OC43 is more similar to HCoV-HKU1 (beta-HCoV) that was recovered in 2005 from adult patients with pneumonia [78]. Their fundamental difference from all other HCoVs is that their virions have two surface projections participating in infection: the common in all HCoVs S protein, and protrusions comprised of hemagglutinin esterase (HE) [79]. In the case of both OC43 and HKU1 the S protein binds to 9-*O*-acetyl-sialic acids, attached to glycoproteins and lipids of the host cell membrane, in order to infect while the HCoV HE protrusions don’t seem to play a vital role [80,81].

The sialoglycan binding site of the S protein is conserved among all CoVs that bind to 9-*O*-acetyl-sialic acids, including the two HCoVs OC43 and HKU1. Sialic acids are important receptors in many human pathogens including Influenza viruses. Specifically, Influenza A/B hemagglutinin esterase (HE) and Influenza C/D hemagglutinin esterase fusion protein (HEF), all use modified sialic acids for infection. Also, it has been proposed that the HE protrusions of OC43 have a phylogenetic relationship with Influenza C HEF, while at the same time act in synergy with the 9-*O*-acetyl-sialic acids-binding domain of the OC43 S protein [79]. It is observed that ligand specificity of HKU1 and OC43 S protein and Influenza C/D HEF is similar, as all recognize 9-*O*-acetylsialic acids through hydrogen bonding with the 9-*O*-acetylcarbonyl moiety and formation of a hydrophobic pocket accommodating the 9-*O*-acetylmethyl group [82]. 

To our knowledge, no study on the antiviral potential of extracts against OC43 and HKU1 exists up to now, while no NPs were ever tested for HKU1 antiviral activity. Nevertheless, several studies exist concerning purified NPs/SMs, with emphasis on isoquinoline alkaloids. Cepharanthine, fangchinoline and tetrandrine isolated from *Stephania tetrandra* (Menispermaceae) have been very recently shown to inhibit OC-43 induced cell death of lung cells, in early stage of infection, and suppress viral replication [83,84]. Additionally, in a recent screening for broad spectrum anti-HCoV agents against NL63, OC43 and MERS, alkaloids lycorine (Table S1) and emetine inhibited viral replication of all strains with EC_50_ below 5 μM [84]. In the same study, the in vivo antiviral activity was established against a lethal intraperitoneal injection of OC43 in female BALB/c mice, where after administration of lycorine for 14 days post-inoculation (15 mg/kg) more than 80% of the mice were still alive [84].

### 3.2. MERS-CoV

A limited number of NPs have been tested for anti-MERS-CoV activity in cell-based infection assays, while their activity has not been associated with a specific target. The majority of the NPs/Sms tested exhibited good activity (EC_50_ < 20 μΜ), nevertheless, SI was found relatively low for most compounds. Alkaloids like emetine (ipecac alkaloids), lycorine (Amaryllidaceae alkaloids), harmine (β-carboline alkaloids), and conessine (steroidal alkaloids) exhibit increased potency with EC_50_ < 5 μΜ. Emetine in particular, was found to inhibit viral S-mediated entry [84] Notably, silvestrol, a flavagline isolated from the tree bark of *Aglaia* sp. (Meliaceae) [85] is the only NP with an EC_50_ in the nanomolar range (1.3 nM), by inhibiting the expression of MERS N-protein and nsp8 [75]. Only one original publication investigates a large panel of secondary metabolites isolated from *Broussonetia papyrifera* (Moraceae), for their ability to inhibit MERS two major proteases PL^pro^ and 3CL^pro^. Limited potency was found for all tested compounds, against both proteases (IC_50_ > 27 μΜ) [86]. Recently, a library containing 502 NPs/SMs of various origin was screened for anti-MERS activity, in a MERS pseudovirus pre- infection assay. After confirmation of the actives in pre- and post- infection assays with MERS, dihydrotanshinone was identified to both block the viral entry by binding to the S protein of MERS, and possibly inhibiting viral replication [87].

### 3.3. SARS-CoV

Limited studies exist examining the anti-SARS-CoV activity of plant extracts, while some promising results of antiviral activity have no follow-up to determine the mechanism of action and specific targets [88,89,90,91,92,93,94]. A study on more than 200 plant extracts against two SARS-CoV viral strains (BJ-001 and BJ-006) revealed that the ethanol cortex extract of *Lycoris radiata* (Amaryllidaceae) inhibited the virus-induced CPE in both strains (EC_50_ 2.4 and 2.1 μΜ, respectively) and had good SI (>350). After fractionation of the active extract, the antiviral activity was attributed to the alkaloid fraction that contains lycorine, although the specific mechanism of action has not been identified [89]. 

The anti-herpes activity of aloe emodin, a known anthraquinone isolated from *Rheum palmatum* (Polygonaceae), has prompted studies for anti-HCoV activity. Aloe emodin has been studied in multiple aspects of viral infection, although no studies of cell-based direct anti-SARS-CoV activity have been performed with the use of a real SARS strain. Aloe emodin is shown to bind the S protein of SARS-CoV thus possibly inhibiting host cell entry [95], inhibit the ion channel formed by the 3a protein of SARS and OC43 strains possibly inhibiting virus release, [96] and very weakly inhibit the activity of SARS 3CL^pro^ [97]. Other examples of NPs/SMs with rather well-defined modes of anti-HCoV action tetra-*O*-galloyl-β-D-glucose, which binds to the S protein of SARS, inhibiting host cell entry of the virus [98], and cyclosporine A and derivatives thereof, which are known to suppress SARS-CoV and MERS-CoV replication by inhibiting cyclophilin A [99].

Notable anti-SARS-CoV activity is attributed to the phenanthraindolizidine alkaloids tylophorine (Figure 3) and tylophorine N-oxide isolated from *Tylophora indica* (Apocynaceae), with EC_50_ 0.018 and 0.34 μΜ, respectively [100]. As shown from published data, natural and semisynthetic pentacyclic triterpenes have shown very good anti-SARS-CoV activity, with betulonic acid being the most active with an EC_50_ of 0.63 μΜ [37,101,102,103]. Interestingly, the 3β-OH analogue of betulonic acid (betulinic acid), had a weak antiviral activity (EC_50_ > 10 μΜ), although it strongly inhibited SARS-CoV 3CL^pro^ (IC_50_ = 10 μΜ), contrary to betulonic acid (IC_50_ > 100 μΜ) [102]. Diterpenoids with various structures have also a significant potency against SARS-CoV, with the abietane diterpene ferruginol isolated from *Chamaecyparis obtusa* (Cupressaceae) showing an EC_50_ of 1.39 μΜ, while the labdane diterpene pinusolidic acid inhibited SARS-CoV infection with an EC_50_ of 4.71 μΜ and an excellent SI (159) [102].

Some other structurally diverse NPs /SMs have shown anti-SARS-CoV activity with EC_50_ close or below 10 μΜ (reserpine, β-yohimbine [37], gallic acid [98], honokiol, forskolin, magnolol [102], luteolin [98]), yet most of the compounds tested exhibited limited activity (EC_50_ > 20 μΜ) [37,91,102,104,105].

Significant efforts towards the discovery of viral cysteine protease inhibitors from NPs/SMs have been made. 3CL^pro^ is inhibited by several NPs with diverse structures such as biflavonoids (amentoflavone, ginkgetin, sciadopitysin, bilobetin) [106], flavonoids (apigenin, quercetin, hyperoside, quercetine L-fucose derivatives, herbacetin, luteolin, pectolinarin, rhoifolin, hesperetin, kazinol A, kazinol B, broussoflavan A, papyriflavonol A, kaempferol, 4-hydroxyisolonchocarpin [86,97,106,107], isolflavones (daidzein) [97], pentacyclic triterpenes (betulinic acid, celastrol, iguesterin, pristimerin, tingenone) [102,108], phytosterols (β-sitosterol) [109], lignans (savinin) [102], indole alkaloids (indican, indigo, indirubin, semisynthetic isatin analogues) [97,110,111], glucosinolates (sinigrin) [97], anthraquinones (emodin, aloe emodin) [109], theaflavins (3-Isotheaflavin-3-gallate, theaflavin-3,3′-digallate) [112], phenanthrenes (crytotanshinone, tanshinone IIa, dihydrotanshinone I, tanshinone I, methyl tanshinonate, tanshinone IIB, rosmariquinone) [113], phlorotannins (dieckol, 7-phloroeckol, 2-phloroeckol, dioxinodehydroeckol, phlorofucofuroeckol A, fucodiphloroethol G) [114], chalcones (broussochalcone B, 3′-(3-methylbut-2-enyl)-3′,4,7-trihydroxyflavane, isoliquiritigenin, broussochalcone A) [86,115], (diarylheptanoids (hirsutenone, hirsutanonol, rubranoside B, rubranoside A, rubranol, oregonin, platyphyllenone, platyphyllonol 5-*O*-β-*D*-xylopyranoside, platyphyllone, curcumin) [102,116], and diarylpropanoids (kazinol F) [86]. Among the NPs tested, the pentacyclic triterpene iguestrin, isolated from the root methanol extract of *Tripterygium regeli* (Celastraceae) was the most active with an IC_50_ of 2.6 μΜ, followed by pristimerin (5.5 μΜ) [108], amentoflavone (8.3 μΜ) [106], and 3-theaflavin-3-gallate (9.8 μΜ) [112]. Notably, semisynthetic diversely substituted analogues of isatin, a small molecule of natural origin derived from the oxidation of the purple dye indigo, were found very active towards 3CL^pro^, with IC_50_ ranging from the lower micromolar range (0.95 μΜ), to 23.5 μΜ [110,111]. 

Similarly, several NPs/SMs inhibit SARS-CoV PL^pro^, like chalcones (bavachalcones, broussochalcones) [86,115,117], cinnamates, [118] coumarins, [117] diarylheptanoids (hirsutenone, curcumin, hirsutanonol, rubranosides A and B, rubranol, oregonin, isoliquiritigenin, papyriflavonol A) [86,116], diarylpropanoids (kazinols) [86], flavonoids (tomentins, quercetin, diplacone, mimulone, kaempferol, hyperoside, etc.) [86,119], isoflavones (corylifol A, neobavaisoflavone, bavachinin) [117], and phenanthrenes (tanshinones, rosmariquinone) [113]. Notably, tanshinone derivatives isolated from *Salvia miltiorrhiza* (Lamiaceae) have been found to exhibit very good inhibitory activity against PL^pro^, with cryptotanshinone having an IC_50_ of 0.8 μΜ [113], while an unusual a chalcone derivative with a hyperoxide group isolated from the ethanol leaves extract of *Angelica keiskei* (Apiaceae) showed an IC_50_ of 1.2 μΜ [115].

Finally, only one study investigates the inhibition of SARS-CoV helicase from NPs. The known flavonoids scutellarein and myricetin were found to inhibit nsp13 with IC_50_ 0.86 and 2.71 μΜ, respectively [120].

Very few NPs or NPs-inspired SMs/SMs, have been investigated for their in vivo anti-HCoV effect. In a 2006 study, several in vitro viral inhibitors were also tested for in vivo efficacy. Chloroquine and phosphate salts, amodiaquine and pentoxifylline were first evaluated in vitro against SARS-CoV replication, with EC_50_ ranging from 1 to 10 μΜ [121]. Chloroquine and amodiaquine are quinoline analogues of quinine, the latter isolated from the bark of *Cinchona* sp. (Rubiaceae), and pentoxifylline is a synthetic analogue of theophylline present in tea and cocoa tee. The in vitro promising results were not verified in the BALB/c SARS-CoV infection model, where all the aforementioned compounds did not reduce the virus titer in the lungs post-infection, in a statistically significant manner [122]. More recently, a study of the effect of compassionate use of hydroxychloroquine or chloroquine in-hospital outcomes for COVID-19 was unable to confirm the benefit of such treatment [123]. The discovery of most of these NPs/SMs was based on high throughput 3CL^pro^ inhibitory assays. For instance, herbacetin, pectolinarin and rhoifolin were found to efficiently block the enzymatic activity of SARS-CoV 3CL^pro^ following a screening of a large flavonoid library against purified 3CL^pro^ using a tryptophan-based fluorescence method to monitor flavonoid-dependent inhibition of proteolysis of a custom-synthesised fluorogenic substrate [124].

Glycyrrhizin (Figure 3) was found to inhibit both SARS-CoV penetration in host cells and virus replication (SI 67). A 2003 study investigating the effect of glycyrrhizin during several stages of infection of Vero cells i.e., during virus absorption, after absorption, and both during and after absorption reported that glycyrrhizin was most effective both during and after adsorption (EC_50_ 300 mg/L) [125]. This study prompted for the development of several glycyrrhizin analogues that showed very potent SARS replication inhibitory activity and, in some cases, SI > 41. For carbamido- analogues of glycyrrhizin, the EC_50_ was significantly low, and that lead to the speculation that those analogues bind to the S protein of SARS through the glucosamine moiety, thus blocking viral entry to the cell [101]. When tested against HCoV-NL63, glycyrrhizin showed no antiviral effect [126]. Glycyrrhizin moderately inhibits infection of Vero cells with porcine epidemic diarrhea virus (PEDV), a coronavirus prevalent in the swine industry. It inhibits viral entry and replication, while virus assembly and viral release remain unaffected. Interestingly, it was found that glycyrrhizin significantly decreases the mRNA of proinflammatory cytokines, namely IL-6, IL-8 and TNF-a, suggesting that it reduces the proinflammatory response of the host cells during viral infection [127]. This reduction in the proinflammatory effects by glycyrrhizin, has been confirmed also with infection of lung epithelial cells with the highly pathogenic H5N1 influenza strain [128]. For SARS, the antiviral mechanism of action of glycyrrhizin is unclear and may include both the interaction with a specific molecular target, and the attenuation of proinflammatory host cell response, as suggested by the previous studies. 

SARS infection inflicts a dysregulation in the immune response of the host and is followed by upregulation of proinflammatory cytokines and activation NF-κB. NPs inhibiting NF-κB, and thus having the potential to diminish the severity of SARS infection, have been studied in mouse models. Caffeic acid phenethyl ester (CAPE) and parthenolide, although having no impact on SARS replication, were found to significantly decrease the levels of proinflammatory cytokines and lung pathology of SARS infected BALB/c mice [129]. Most importantly, treatment with these drugs that inhibited NF-κB activation led to reduction in inflammation and lung pathology in both SARS-CoV-infected cultured cells and mice and significantly increased mouse survival after SARS-CoV infection. These data indicated that activation of the NF-κB signaling pathway represents a major contribution to the inflammation-induced after SARS-CoV infection and that NF-κB inhibitors are promising antivirals in infections caused by SARS-CoV and potentially other pathogenic human coronaviruses. 

### 3.4. SARS-CoV-2

#### 3.4.1. Virtual Screening Approaches

Crystallography of HCoV enzymes and reports on their potential ligands, have assisted computational methods (docking-scoring calculations of protein-ligand interactions using similarity to known actives) to discover SARS-CoV-2 inhibitors [27]. Virtual screening is extensively employed for the moment, to identify potential ligands from SMs and in-house NPs libraries [130,131,132].

Regarding 3CL^pro^, recent efforts, have identified potential inhibitors among: alkaloids [133,134,135,136,137,138], and especially indole alkaloids [139,140,141,142,143,144]; terpenoids [138], and especially diterpenes [133,145]; sesquiterpenes [134,146,147], sesquiterpene lactones [148], and triterpenes [133,136,140,149]; anthocyanins [150,151] and proanthocyanidins [152,153]; ellagitannins [153,154]; flavonoids [133,137,140,144,147,148,152,155,156,157,158,159,160,161], biflavonoids [162], and macrocyclic flavonoids [148]; isoflavones [144,148]; chalcones [163,164]; lignans [133,134]; coumarins [159]; caffeic acid esters and cinnamates [144,153,165,166]; stilbenes [148]; diarylheptanoids [165,167]; polyketides [138]; quinones [168]; oligosaccharides [157]; depsipeptides [169]; and xanthones [164].

In a more limited number of studies, potential affinity to PL^pro^ has been suggested for flavonoids and biflavonoids [133]; chalcones [163]; caffeic acid esters [133]; terpenoids and sesquiterpene lactones [133,138]; polyketides [138]; and alkaloids [133,138]. 

The active site of RdRp has been also investigated as a possible target of inhibition finding multiple possible ligands, such as flavonoids, xanthones [133], polyketides [138], terpenes and sesquiterpene lactones [133,138], and alkaloids [138,143]. Finally, alkaloids, polyketides and terpenes have shown affinity for non-structural protein 15 (nsp15) of SARS-CoV-2 [138], while several NPs have been investigated as potential ligands to virulence factors Nsp1 (suppresses type-I IFN expression), Nsp3c (promotes replication of the viral genome and transcription of viral mRNA) and ORF7a (inhibits virus restriction) [133].

Homology modeling is a tool that was also used for the identification of NPs that interfere with the S protein interface with the human receptor ACE2. The only NP that was identified through virtual screening to potentially interfere with ACE2-mediated host cell entry, was hesperidin [170], although selected flavonoids and epigallocatechins have shown an affinity for the S protein. Recent research indicated that the S protein of SARS-CoV-2 contains a furin-like cleavage site that is absent from SARS-CoV, pointing to the need of developing specific furin inhibitors [17]. This is a new finding that is currently under intensive investigation [171]. 

It has been demonstrated that the serine protease TMPRSS2 is needed for S protein priming and host cell entry [11]. Virtual screening of natural SM libraries against a TMPRSS2 structure built by homology modeling detected several potential protease inhibitors, including norsesquiterpenes, diterpenes and xanthones, [133,164,172] alkaloids, [173] chalcones, [164] coumarins, [173] and flavonoids. [174] Interestingly, SARS-CoV infectivity is reportedly associated TMPRSS2 expression levels, which are induced by androgens and suppressed by estrogens, implying that natural estrogen receptor agonists (e.g., genistein) and androgen receptor antagonists (e.g., atraric acid, indole-3-carbinol, niphatenone B) could impact SARS-CoV-2 infection [175,176,177,178]. 

#### 3.4.2. Actual Screening of NPs for Anti-SARS-CoV-2 Activity

The first results from the physical screening of NPs for anti-SARS-CoV-2 using host cells and/or viral enzymes have been published very recently. A high throughput cell-based screening assay was established by Zhang et al. [179] according to which the effect of potential inhibitors in the CPE in Vero-E6 cells is assessed in the entire viral cell cycle. Known inhibitors were used as positive controls (remdesivir, chloroquine, neutralizing human antibody CB62 and IFN-α), while multiple concentrations of DMSO in the culturing media was used in order to determine potential solvent effects on CPE. A library of 1058 compounds was screened, in a two-level assay: the first screening revealed 30 hits (>50% protection from CPE), 17 of which have never been associated with SARS-CoV-2 through different assays. (Table 1) Further evaluation showed that viral propagation was inhibited in a dose-dependent manner, while EC_50_ values ranged between 0.011 and 11.03 μΜ. In an attempt to identify possible mechanisms of action of active compounds bufalin and digoxin, it was postulated that those compounds target the ion transport function of Na+/K+-ATPase, and intracellular ion homeostasis [179], with the most potent inhibitor represented by the quassinoid derivative bruceine A (Table 1) that displayed an EC_50_ value of 0.011 μΜ and a SI of 2854. 

The only existing study for associating anti-SARS-CoV-2 activity of an extract with isolated components, concerns the investigation of plant biodiversity of Thailand. Three extracts from respective plants are reported to have viral infection inhibition, namely *Andrographis paniculata* (Acanthaceae), *Zingiber officinale* (Zingiberaceae), and *Boesenbergia rotunda* (Zingiberaceae), are reported to display viral infection inhibition. The extract of *B. rotunda* and the isolate panduratin A (Table 1) suppressed SARS-CoV-2 infectivity in Vero E6 cells with EC_50_ of 3.62 μg/ mL and 0.81 μM, respectively [180].

Concerning the inhibition of SARS-CoV-2 main protease 3CL^pro^, only three publications exist for the moment, reporting the flavonoids baicalin and baicalein, as potent inhibitors with an IC_50_ of 6.41 and 0.94 μΜ, respectively, as well as inhibitors of SARS-CoV-2 replication in Vero E6 cells [181]. Baicalin has also been investigated for the in vitro propagation of SARS-CoV in Vero-E6 cells, though no significant activity was found (EC_50_ > 100 μM) [105]. Additionally, the labdane diterpene andrographolide and a semisynthetic derivative displayed inhibitory activity against 3CL^pro^ [182], while tannic acid was found active with an IC_50_ of 2.1 μΜ [183].

Finally, the well-known flavonoid quercetin (Figure 3) has been proposed as a SARS-CoV-2 3CL^pro^ inhibitor (Ki = 7.4 μM), while molecular simulations showed that it binds to the active site of the enzyme [184]. Flavonoids have been highlighted in the past as HCoVs protease inhibitors. Quercetin itself weakly inhibits SARS-CoV 3CL^pro^ (IC_50_ 23.8 μΜ), and shows no inhibition in MERS-CoV 3CL^pro^, while it is reported to potently inhibit SARS-CoV PL^pro^ (IC_50_ 8.6 μΜ) [106]. Although these results may seem promising, quercetin has not been tested in cell viral infection assays, and additionally, like many dietary flavonoids and polypenols, has poor oral bioavailability [185]. 

NPs have not yet been investigated thoroughly for anti-HCoV activity. Nevertheless, NPs such as the Nobel Prize awarded antimalarial drug artemisinin, isolated from *Artemisia annua* (Asteraceae), have shown notable bioactivity against viruses of the Herpesviridae family (e.g., herpes simplex virus type 1 and Epstein-Barr virus), hepatitis B virus, hepatitis C virus, and bovine viral diarrhea virus [186].

#### 3.4.3. Host Interactions and Future Prospects

Compounds targeting host cell components may help to treat SARS-CoV-2 infection. It has been reported that certain NPs (cholesterol, β-sitosterol, betulinic acid, hopane and glycyrrhizin), may reduce SARS-CoV-2 infectivity by inhibiting lipid-dependent attachment of the virus to host cells [187,188]. It is also reported that 25-hydroxy-cholesterol shows broad antiviral activity by blocking membrane fusion in the viral infection stage [189]. A recent study of SARS-CoV-2 human protein interactome reported that as many as 332 human proteins are intercepted by SARS-CoV-2 proteins, with several of these interceptions potentially targeted by NPs/SMs already approved by FDA or in preclinical or clinical development [190].

This study [190] revealed that the SARS-CoV-2 infection, replication and biogenesis program interfered with host components involved in DNA replication, regulation of gene expression, RNA processing and translation, protein expression and ubiquitination, ER/Golgi function and vesicle trafficking, nuclear transport, mitochondrial import receptors, extracellular matrix cytoskeletal function, IFN signaling, NF-kB-mediated inflammatory response and innate immune response and lipid modification. Some of these interactions could be targeted by NPs/SMs. For instance, the non-structural protein 6 (Nsp6) component of the viral replication complex may interfere with the function of vacuolar ATPase ATP6AP1 and the endoplasmic reticulum endomembrane compartments to favor coronavirus replication, an interference that might be inhibited by the macrolide antibiotic bafilomycin A1, an ATPase inhibitor in preclinical development. With regard to host proteases, the interactome study reported that 3CL^pro^ may interfere with histone deacetylase 2-mediated inflammation and interferon response to SARS-CoV-2 infection in a manner inhibited by the fungal metabolite apicidin, a histone deacetylase inhibitor in preclinical development [191]. 

Virus-encoded helicase interacts with the centrosomal protein CEP250 and the interaction might be targeted by the natural polyketide WDB002. Virus-encoded 3′-5′ exonuclease interacts with the purine biosynthesis enzyme IMPDH2, a target of cyclophilin A, which in turn is involved in the packaging of the viral capsid; the IMPDH2-CypA interaction is modulated by the natural product sanglifehrin A [190].

At the beginning of the pandemic, the use of kinase inhibitors was proposed as a therapeutic strategy against COVID-19. Some kinase inhibitors, such as imatinib, dasatinib and trametinib, that have been shown to inhibit viral replication in vitro [192], may rely on an indirect inhibition of TMPRSS2 function. Indeed, although there is no demonstrated association between TMPRSS2 and kinase inhibition, it has been postulated that kinase inhibition could result in the inhibition of TMPRSS2 function, localization, or activity, and the observed blocking of the infection. Abl kinase and the ERK/MAPK, and PI3K/AKT/mTOR signaling pathways have been shown to play a role in the relevant MERS-CoV and SARS-CoV infection [192,193]. Imatinib, a BCR-Abl kinase inhibitor, is under clinical investigation for having beneficial results in hospitalized adults with COVID-19 [194], although it has been shown that it has no antiviral activity [195]. 

The role of kinases in the pathology of COVID-19, and especially in the inflammatory responses, is investigated intensively. In a recent study [196] it was found a causal link between life-threatening COVID-19 and high expression of *TYK2*, the gene encoding Tyrosine Kinase 2, a member of the JAK family kinases. *TYK2* abnormalities have been established in several autoimmune diseases such as systemic lupus [197], rheumatoid arthritis [198], and multiple sclerosis [199]. Small molecule TYK2 inhibitors are under clinical trials for their safety and efficacy in psoriasis [200,201], and other autoimmune diseases associated with inflammation [202]. The natural product parthenolide is shown to inhibit all three Janus kinases (JAK1, JAK2 and TYK2), thus inhibiting STAT3 signaling [203]. Interestingly, parthenolide and caffeic acid phenyl ethyl ether (CAPE), were tested as NF-κΒ inhibitors in a mouse in vivo model of SARS-CoV, and found to significantly reduce lung inflammation and pathology [129].

## 4. Conclusions

Natural products have played an active and important role in drug discovery up until today. Nevertheless, their valorization as antiviral agents remains limited. It is indicative that antiviral NP research has peaked only around imminent threats, as happened with SARS-CoV. Our review of the bibliography covering the study of NPs against HCoVs, revealed that there are many possibilities in examining more thoroughly available NPs in order to discover new antiviral agents. NP databases and NP libraries with physical samples available for bioactivity screening may have shortened the time needed for a compound to reach bioactivity evaluation, but as shown from the literature survey, tested compounds tend to revolve around very common and not very diverse structures. In terms of anti HCoV activity and NPs, the available literature is limited in order to draw sound conclusions about structure-activity relationships. Nevertheless, the data show that there is a trend for alkaloids, triterpenoids/triterpene saponins, and polyhydroxylated flavonoids. HCoVs and NPs research has been fragmented up to this point, driven by states of emergency, such as the emergence of SARS-CoV in 2002, and the current threat of SARS-CoV-2. Although there are a number of published results, there is a lack of systematic investigation of NPs showing promising activity, and this needs to be addressed by research efforts defining the mechanism of action of these compounds. Under this scope, compounds based on NPs scaffolds with higher potency, more favorable physicochemical properties, and diminished toxicity, can be designed, thus providing a multidisciplinary approach that antiviral discovery needs. NPs offer great chemodiversity that needs to be further exploited, especially under the current pressure of this global pandemic. 

## Figures and Tables

**Table 1:** Antiviral activity of selected NPs against HCoV-NL63, HCoV-OC43, MERS-CoV, SARS-CoV, and SARS-CoV-2.

Structure	Name	Source	Target	Assay		Ref.
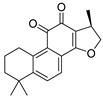	Cryptotanshinone	*Salvia miltiorrhiza* Lamiaceae	SARS-CoV	Enzyme inhibition (IC_50_)	PL^pro^ 0.8 μΜ, 3CL^pro^ 226.7 μΜ	[113]
			SARS-CoV-2	CPE inhibition (EC_50_)	5.024 μΜ	[179]
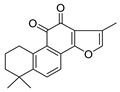	Tanshinone IIA	*Salvia miltiorrhiza* Lamiaceae	SARS-CoV	Enzyme inhibition (IC_50_)	PL^pro^ 1.6 μΜ, 3CL^pro^ 89.1 μΜ	[113]
			SARS-CoV-2	CPE inhibition (EC_50_)	<11 μΜ	[179]
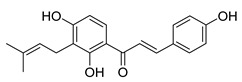	Isobavachalcone	*Psoralea* sp. Fabaceae	SARS-CoV	Enzyme inhibition (IC_50_)	PL^pro^ 7.3 μΜ	[117]
			SARS-CoV-2	CPE inhibition (EC_50_)	<11 μΜ	[179]
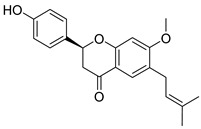	Bavachin	*Psoralea* sp. Fabaceae	SARS-CoV	Enzyme inhibition (IC_50_)	PL^pro^ 38.4 μΜ	[117]
			SARS-CoV-2	CPE inhibition (EC_50_)	<11 μΜ	[179]
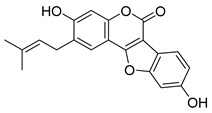	Psoralidin	*Psoralea* sp. Fabaceae	SARS-CoV	Enzyme inhibition (IC_50_)	PL^pro^ 4.2 μΜ	[117]
			SARS-CoV-2	CPE inhibition (EC_50_)	<11 μΜ	[179]
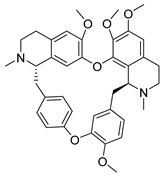	Tetrandrine	*Stephania tetrandra* Menispermaceae	HCoV-NL63	CPE inhibition (EC_50_)	2.05 μΜ	[84]
			HCoV-OC43		0.29 μΜ/0.33 μΜ	[83,84]
			MERS-CoV		12.68 μΜ	[84]
			SARS-CoV-2	CPE inhibition (EC_50_)	<11 μΜ	[179]
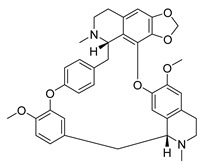	Cepharanthine	*Stephania tetrandra* Menispermaceae	HCoV-OC43	CPE inhibition (EC_50_)	0.83 μΜ	[83]
			SARS-CoV-2	CPE inhibition (EC_50_)	<11 μΜ	[179]
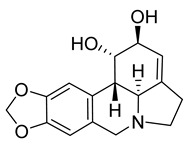	Lycorine	*Lycoris* sp. Amaryllidaceae	HCoV-NL63	CPE inhibition (EC_50_)	0.47 μΜ	[84]
			HCoV-OC43		0.15 μΜ	
			MERS-CoV		1.63 μΜ	
			SARS-CoV		169.8 μΜ	[89]
			SARS-CoV-2	CPE inhibition (EC_50_)	<11 μΜ	[179]
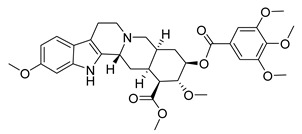	Reserpine	*Rauvolfia serpentina* Apocynaceae	SARS-CoV	CPE inhibition (EC_50_)	3.4 μΜ	[37]
			SARS-CoV-2	CPE inhibition (EC_50_)	<11 μΜ	[179]
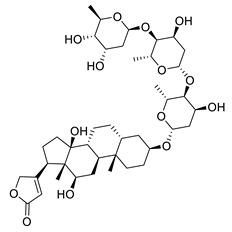	Digoxin	*Digitalis* sp. Plantaginaceae	SARS-CoV-2	CPE inhibition (EC_50_)	0.1541 μΜ	[179]
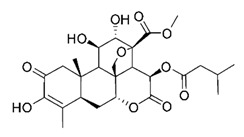	Bruceine A	*Brucea javanica* Simaroubaceae			0.011 μΜ	
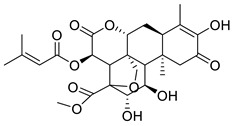	Brusatol	*Brucea javanica* Simaroubaceae			0.0492 μΜ	
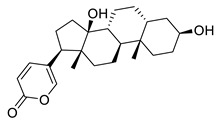	Bufalin	Toad venom Bufonidae			0.018 μΜ	
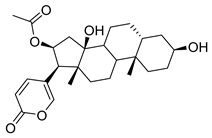	Bufotalin	Toad venom Bufonidae			0.0259 μΜ	
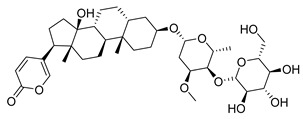		Toad venom Bufonidae			0.0657 μΜ	
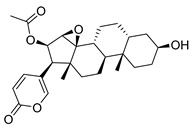	Cinobufagin	Toad venom Bufonidae			0.018 μΜ	
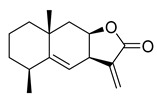	Alantolactone	*Inula helenium* Asteraceae			1.724 μΜ	
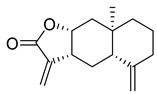	Isoalantolactone	*Inula helenium* Asteraceae			1.483 μΜ	
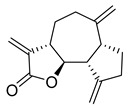	Dehydrocostus lactone	*Saussurea costus* Asteraceae			2.322 μΜ	
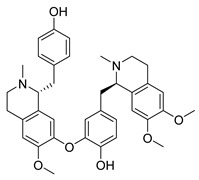	Liensinine	*Nelumbo nucifera* Nelumbonaceae			2.537 μΜ	
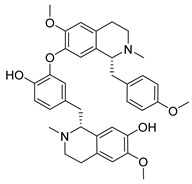	Isoliensinine	*Nelumbo nucifera* Nelumbonaceae			1.615 μΜ	
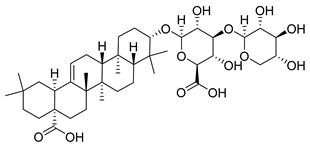	Momordinic	*Bassia scoparia* Amaranthaceae			3.529 μΜ	
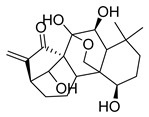	Oridonin	*Isodon* sp. Lamiaceae			1.462 μΜ	
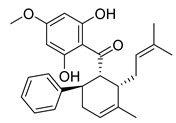	Panduratin A	*Boesenbergia rotunda* Zingiberaceae	SARS-CoV-2	CPE inhibition (EC_50_)	0.81 μΜ	[180]

## Supplementary Materials

Table S1: Natural products tested for anti-HCoV activity in cell based assays; Table S2: Natural products tested for HCoV viral enzyme inhibition. Table S3: Inhibitory activity of extracts against the main protease of SARS-CoV, 3CL^pro^; Inhibition of HCoV enzymes from natural and nature-derived products; Table S4: Inhibition of HCoV enzymes from natural and nature-derived products.
