# Supplementary material for: Natural and Nature-Derived Products Targeting Human Coronaviruses
Source: Molecules. 2021 Jan 16;26(2):448. doi: 10.3390/molecules26020448 (PMC7831024; doi:10.3390/molecules26020448)
Supplement: Supplementary file 1 [file molecules-26-00448-s001.pdf]

Supporting Information

Natural and nature-derived products targeting human coronaviruses

Konstantina Vougogiannopoulou [1], Angela Corona [2], Enzo Tramontano [2], Michael N. Alexis [3], Alexios-Leandros Skaltsounis [1*].

[1] Department of Pharmacognosy and Natural Products Chemistry, Faculty of Pharmacy, National and Kapodistrian University of Athens, Panepistimiopolis Zografou, 15771 Athens, Greece; nadia_voug@pharm.uoa.gr, skaltsounis@pharm.uoa.gr

[2] Department of Life and Environmental Sciences University of Cagliari, Biomedical Section, Laboratory of Molecular Virology, E block. Cittadella Universitaria di Monserrato, SS554 - 09042 Monserrato (CA) Italy; angela.corona@unica.it, tramon@unica.it

[3] Molecular Endocrinology Team, Inst of Chemical Biology, National Hellenic Research Foundation (NHRF), 48 Vassileos Constantinou Ave., 11635 Athens, Greece; mnalexis@eie.gr

Table of Contents

TABLE S 1 Plant extracts tested for anti HCoV activity in various strains 1

TABLE S 2 Pure natural products and nature derived compounds tested for anti HCoV activity in various strains 2

TABLE S 3 Inhibitory activity of extracts against the main protease of SARS-CoV, 3CL^{pro}. 19

TABLE S 4 Inhibition of HCoV enzymes from natural and nature-derived products. 20

TABLE S 1 Plant extracts tested for anti HCoV activity in various strains

Plant	Family	Plant part / extract	Viral strain	Host cells, time	CC_{50} (μg/mL)	EC50 (μg/mL)	Ref.
Stevia sp.	Asteraceae	Leaves	HCoV-229E	Vero, 72h	-	lg TCID50, 0.33	10.1007/s11094-009-0270-7
Pelargonium sidoides	Geraniaceae	Roots/EtOH	HCoV-229E	Caco-2	>100	44.5	10.1016/j.phymed.2010.09.008
Strobilanthes cusia	Acanthaceae	Leaves/MeOH	HCoV-NL63	LLC-MK2, 48h	>100	0.64	10.3390/biom10030366
Cibotium barometz	Cibotiaceae	Roots/water	SARS-CoV	Vero-E6, 72h	>500	8.42	10.1016/S2225-4110(16)30055-4
Gentiana scabra	Gentianaceae	Roots/water	SARS-CoV	Vero-E6, 72h	>500	8.7	
Dioscorea batatas	Dioscoreaceae	Tubers/water	SARS-CoV	Vero-E6, 72h	>500	8.06	
Cassia tora	Fabaceae	Seeds/water	SARS-CoV	Vero-E6, 72h	>500	8.43	
Taxillus chinensis	Loranthaceae	Aerial parts/water	SARS-CoV	Vero-E6, 72h	>500	5.39	
Lycoris radiata	Amaryllidaceae	Stem cortex/EtOH	SARS-CoV	Vero-E6, 72h	886.6	2.40	10.1016/j.antiviral.2005.02.007
Artemisia annua	Asteraceae	Aerial/EtOH	SARS-CoV	Vero-E6, 72h	1053	34.5	
Pyrrosia lingua	Polypodiaceae	Leaves/CDCl3	SARS-CoV	Vero-E6, 72h	2378	43.2	
Lindera aggegate	Lauraceae	Roots/EtOH	SARS-CoV	Vero-E6, 72h	1374	88.2	
Toona sinensis	Meliaceae	Leaves/water	SARS-CoV	Vero, 72h	>500	30	10.1016/j.jep.2008.07.048
Cinnamomum verum	Lauraceae	Bark/water-EtOH	SARS-CoV	Vero-E6, 72h	180	7.8	10.1016/j.antiviral.2009.02.001
Cupressus sempervirens	Cupressaceae	Cones/EtOH	SARS-CoV	Vero	-	lg TCID50, 4.3	10.3166/phyto-2018-0064
Andrographis paniculata	Acanthaceae	-	SARS-CoV-2	Vero-E6, 72h	-	68.29% inhibition	10.21203/rs.3.rs-32489/v1
Zingiber officinale	Zingiberaeae	-	SARS-CoV-2	Vero-E6, 72h	-	99.97% inhibition	
Boesenbergia rotunda	Zingiberaeae	-	SARS-CoV-2	Vero-E6, 72h	-	99.95% inhibition	

TABLE S 2 Pure natural products and nature derived compounds tested for anti HCoV activity in various strains

**Trivial name**	**Structure**	**Class of compounds**	**Plant**	**Viral strain**	**Host cells, incubation time**	**CC_{50} (µM)**	**EC_{50} (µM)**	**Ref. (DOI)**
8*β*-hydroxyabieta-9(11),13-dien-12-one)	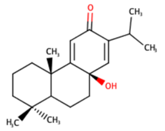	Abietane diterpenes	*Chamaecyparis obtusa*	SARS-CoV	-	>750	1.47	10.1021/jm070295s
7*β*-hydroxydeoxycryptojaponol	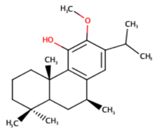	Pentacyclic triterpenes	*Cryptomeria japonica*	SARS-CoV	-	127	1.15	10.1021/jm070295s
3*β*,12-diacetoxyabieta-6,8,11,13- tetraene	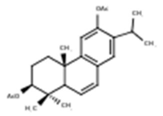	Abietane diterpenes	*Juniperus formosana*	SARS-CoV	-	303.3	1.57	10.1021/jm070295s
6,7-dehydroroyleanone	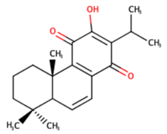	Abietane diterpenes	*Chamaecyparis obtusa*	SARS-CoV	-	89.7	5.55	10.1021/jm070295s
Dehydroabieta-7-one	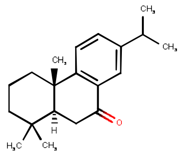	Abietane diterpenes	*Chamaecyparis obtusa*	SARS-CoV	-	305.1	4	10.1021/jm070295s
Ferruginol	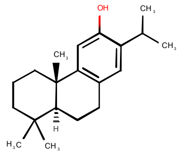	Abietane diterpenes	*Chamaecyparis obtusa*	SARS-CoV	-	80.4	1.39	10.1021/jm070295s
Lycorine		Amaryllidaceae alkaloids	-	HCoV-NL63	LLC-MK2, 72h	3.81	0.47	10.1128/JVI.00023-19

Trivial name	Structure	Class of compounds	Plant	Viral strain	Host cells, incubation time	CC_{50} (μM)	EC_{50} (μM)	Ref. (DOI)
	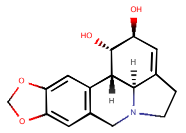			HCoV-OC43	BHK-21, 72h	4.37	0.15	
				MERS-CoV	Vero-E6, 72h	3.14	1.63	
			Lycoris radiata	SARS-CoV	Vero-E6, 72h	>1000	169.8	10.1016/j.antiviral.2005.02.007
Dehydrodiisoeugenol	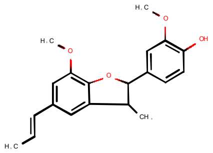	Benzofurans	*Aristolochia taliscana*	SARS-CoV-2	Vero-E6, 72-96h	>100	10.29	10.1038/s41392-020-00343-z
Amiodarone	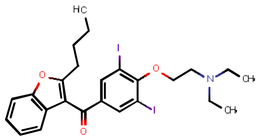	Benzofurans	*Nature derived*	SARS-CoV	Vero, 24h	-	TCID50 below LOD	10.1165/rcmb.2007-0217OC
Berbamine	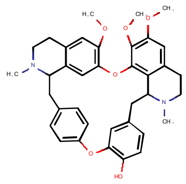	Bisbenzylisoquinoline alkaloids	-	MERS-CoV	Vero-E6, 72h	>20	13.14	10.1128/JVI.00023-19
Cepharanthine	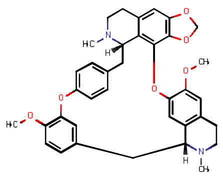	Bisbenzylisoquinoline alkaloids	*Stephania tetrandra*	HCoV-OC43	MRC-5, 72h	11.26	0.83	10.3390/biom9110696

Trivial name	Structure	Class of compounds	Plant	Viral strain	Host cells, incubation time	CC_{50} (µM)	EC_{50} (µM)	Ref. (DOI)
Liensinine	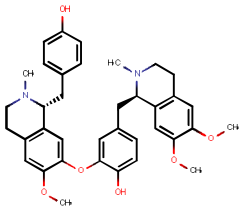	Bisbenzylisoquinoline alkaloids	*Nelumbo nucifera*	SARS-CoV-2	Vero-E6, 72-96h	25.4	2.537	10.1038/s41392-020-00343-z
Isoliensinine	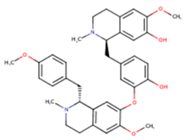	Bisbenzylisoquinoline alkaloids	*Nelumbo nucifera*	SARS-CoV-2	Vero-E6, 72-96h	40	1.615	10.1038/s41392-020-00343-z
Fangchinoline	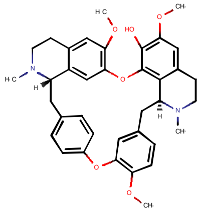	Bisbenzylisoquinoline alkaloids	*Stephania tetrandra*	HCoV-OC43	MRC-5, 72h	11.54	1.01	10.3390/biom9110696
Tetrandrine	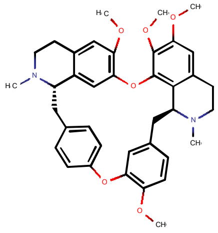	Bisbenzylisoquinoline alkaloids	-	HCoV-NL63	LLC-MK2, 72h	>20	2.05	10.1128/JVI.00023-19
			-	HCoV-OC43	BHK-21, 72h	>20	0.29	10.1128/JVI.00023-19
			Stephania tetrandra	HCoV-OC43	MRC-5, 72h	13.41	0.33	10.3390/biom9110696
			-	MERS-CoV	Vero-E6, 72h	>20	12.68	10.1128/JVI.00023-19
α-cadinol	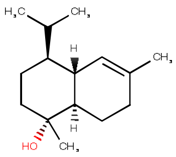	cadinane sesquiterpenoids	*Chamaecyparis obtusa*	SARS-CoV		76.4	4.44	10.1021/jm070295s
4′-Hydroxychalcone		Chalcones	*Nature derived*	HCoV-NL63	LLC-MK2, 72h	>20	7.25	10.1128/JVI.00023-19

**Trivial name**	**Structure**	**Class of compounds**	**Plant**	**Viral strain**	**Host cells, incubation time**	**CC_{50} (μM)**	**EC_{50} (μM)**	**Ref. (DOI)**
	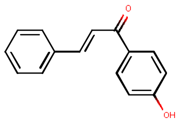			HCoV-OC43	BHK-21, 72h	>20	1.52	
				MERS-CoV	Vero-E6, 72h	>20	10.23	
Coniferyl aldehyde	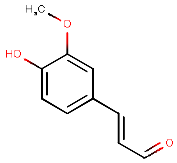	Cinnamates	-	SARS-CoV-2	Vero-E6, 72-96h	>40	11.03	10.1038/s41392-020-00343-z
Leptodactylone	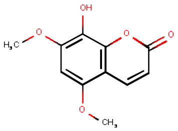	Coumarins	*Boenninghausenia sessilicarpa*	SARS-CoV	Vero-E7	Cytotoxicity 17%	Pathological changes >50-75%	10.1080/10286020500382397
Rutamarin	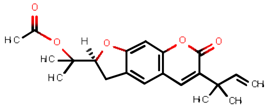	Coumarins	*Boenninghausenia sessilicarpa*	SARS-CoV	Vero-E6	Cytotoxicity 41%	Pathological changes >25-50%	10.1080/10286020500382397
Curcumin	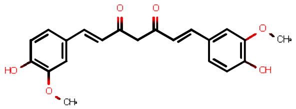	Diarylheptanoids	*Cryptomeria japonica*	SARS-CoV		>250	>10	10.1021/jm070295s
Panduratin A	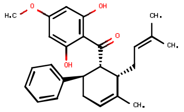	Diarylheptanoids	*Boesenbergia rotunda*	SARS-CoV-2	Vero-E6, 48h	14.71	0.81	10.21203/rs.3.rs-32489/v1
Panduratin A				SARS-CoV-2	Vero-E6, 72-96h	-	2.04	

Trivial name	Structure	Class of compounds	Plant	Viral strain	Host cells, incubation time	CC_{50} (µM)	EC_{50} (µM)	Ref. (DOI)
Dihydrotanhsinone	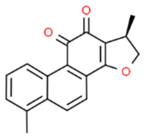	Diterpenes - phenanthrenes		MERS-CoV	Vero-E6	-	pre-attachement, 5.5 logTCID50	10.1016/j.ijantimicag.2018.05.003
				MERS-CoV	Vero-E6	-	post-attachment, 1.8 logTCID50	
Cryptotanshinone	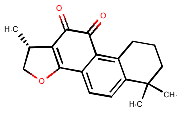	Diterpenes - phenanthrenes	*Salvia miltiorrhiza*	SARS-CoV-2	Vero-E6, 72-96h	>100	5.024	10.1038/s41392-020-00343-z
Oridonin	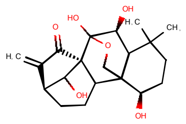	Diterpenes	*Isodon*	SARS-CoV-2	Vero-E6, 72-96h	>40	1.462	10.1038/s41392-020-00343-z
Silvestrol	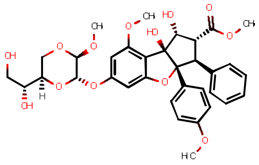	Flavaglines	*Aglaia sp.*	HCoV-229E	PBMCs, 24h	>1	0.0028	10.1016/j.antiviral.2017.12.010
				HCoV-229E	MRC-5, 24h	>3330	0.003	
				HCoV-229E	Huh-7, 24h	0.003	0.04	
				MERS-CoV	MRC-5, 24h	>10	0.0013	
Baicalin		Flavonoids	*Scutellaria sp.*	SARS-CoV	Vero-E6, 48h	>224	224	10.1016/j.jcv.2004.03.003

**Trivial name**	**Structure**	**Class of compounds**	**Plant**	**Viral strain**	**Host cells, incubation time**	**CC_{50} (μM)**	**EC_{50} (μM)**	**Ref. (DOI)**
	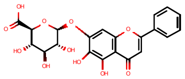			SARS-CoV	fRhK4, 48h	>224	28	
Luteolin	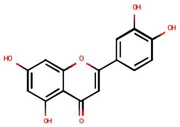	Flavonoids	*Galla chinensis*	SARS-CoV	Vero-E6, 36h	150	10.6	10.1128/jvi.78.20.11334-11339.2004
tetra-O-galloyl-*β*-D-glucose	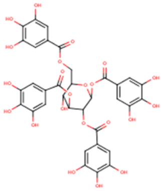	Gallic acid derivatives	*Galla chinensis*	SARS-CoV	Vero-E6, 36h	108	4.5	10.1128/jvi.78.20.11334-11339.2004
Harmine	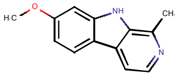	Indole alkaloids	-	HCoV-NL63	LLC-MK2, 72h	>20	13.46	10.1128/JVI .00023-19
				HCoV-OC43	BHK-21, 72h	>20	1.9	
				MERS-CoV	Vero-E6, 72h	>20	4.93	
Indigodole B	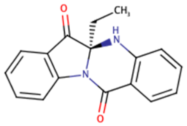	Indole alkaloids	*Strobilanthes cusia*	HCoV-NL63	LLC-MK2, 48h	>400	2.6	10.3390/biom10030366

Trivial name	Structure	Class of compounds	Plant	Viral strain	Host cells, incubation time	CC50 (µM)	EC50 (µM)	Ref. (DOI)
Tryptanthrin	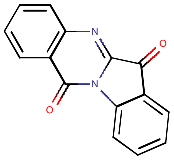	Indoloquinazoline alkaloids	*Strobilanthes cusia*	HCoV-NL63	LLC-MK2, 48h	>400	1.52	10.3390/biom10030366
α-yohimbine	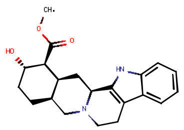	Indoloquinazoline alkaloids	*Aspidosperma sp.*	SARS-CoV	Vero-E6, 72h	-	100	10.1073/pnas.0403596101
β-yohimbine	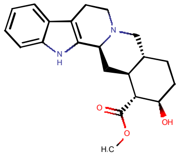	Indoloquinazoline alkaloids	*Aspidosperma sp.*	SARS-CoV	Vero-E6, 72h	-	10	10.1073/pnas.0403596101
Yohimbine	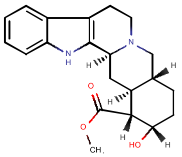	Indoloquinazoline alkaloids	*Aspidosperma sp.*	SARS-CoV	Vero-E6, 72h	-	100	10.1073/pnas.0403596101
Emetine	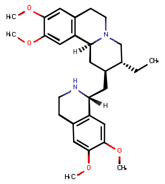	Ipecac alkaloids	-	HCoV-NL63	LLC-MK2, 72h	3.63	1.43	10.1128/JVI.00023-19
				HCoV-OC43	BHK-21, 72h	2.69	0.3	
				MERS-CoV	Vero-E6, 72h	3.08	0.34	
Berbamine		Isoquinoline alkaloids	-	HCoV-NL63	LLC-MK2, 72h	>20	9.46	10.1128/JVI.00023-19

Trivial name	Structure	Class of compounds	Plant	Viral strain	Host cells, incubation time	CC_{50} (µM)	EC_{50} (µM)	Ref. (DOI)
	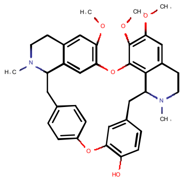			HCoV-OC43	BHK-21, 72h	>20	1.48	
Forskolin	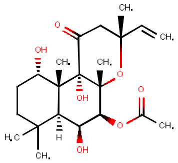	Labdane diterpenes	*Cryptomeria japonica*	SARS-CoV	-	674	7.5	10.1021/jm070295s
Pinusolidic acid	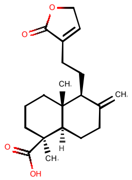	Labdane diterpenes	*Chamaecyparis obtusa*	SARS-CoV	-	>750	4.71	10.1021/jm070295s
Honokiol	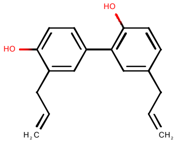	Lignans	*Cryptomeria japonica*	SARS-CoV	-	88.9	6.5	10.1021/jm070295s
Magnolol	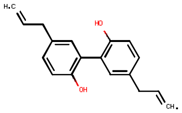	Lignans	*Cryptomeria japonica*	SARS-CoV	-	68.3	3.8	10.1021/jm070295s

**Trivial name**	**Structure**	**Class of compounds**	**Plant**	**Viral strain**	**Host cells, incubation time**	**CC_{50} (µM)**	**EC_{50} (µM)**	**Ref. (DOI)**
Savinin	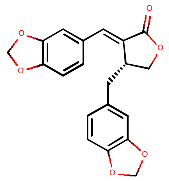	Lignans	*Chamaecyparis obtusa*	SARS-CoV	-	>750	1.13	10.1021/jm070295s
Tacrolimus	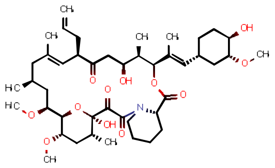	Macrolides	*Streptomyces tsukubaiensis*	HCoV-229E	Huh-7, 48h	-	5.4	10.1016/j.virusres.2012.02.002
			Streptomyces tsukubaiensis	HCoV-NL63	CaCo-2, 48h (PCR)	-	5.1	
Papaverine	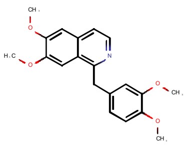	Opium alkaloids	-	HCoV-NL63	LLC-MK2, 72h	11.71	7.32	10.1128/JVI.00023-19
				HCoV-OC43	BHK-21, 72h	12.11	1.61	
				MERS-CoV	Vero-E6, 72h	11.98	9.45	
α-hederin	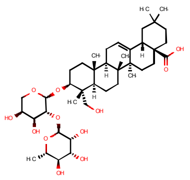	Pentacyclic triterpenes	*Nigella sativa*	SARS-CoV	Vero-E6, 72h	-	10	10.1073/pnas.0403596101
18β-glycyrrhetinic acid derivative 1	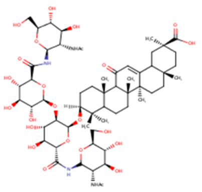	Pentacyclic triterpenes	*Nature derived*	SARS-CoV	Vero-E6, 72h	>3000	40	10.1021/jm0493008

Trivial name	Structure	Class of compounds	Plant	Viral strain	Host cells, incubation time	CC_{50} (µM)	EC_{50} (µM)	Ref. (DOI)
18β-glycyrrhetinic acid derivative 10	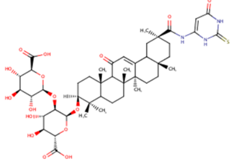	Pentacyclic triterpenes		SARS-CoV	Vero-E6, 72h	250	50	
18β-glycyrrhetinic acid derivative 11	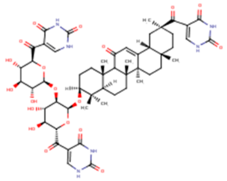	Pentacyclic triterpenes		SARS-CoV	Vero-E6, 72h	15	5	
18β-glycyrrhetinic acid derivative 12	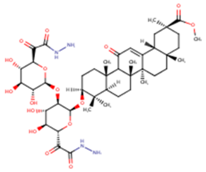	Pentacyclic triterpenes		SARS-CoV	Vero-E6, 72h	66	16	
18β-glycyrrhetinic acid derivative 2	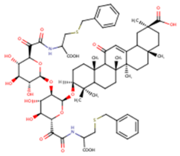	Pentacyclic triterpenes		SARS-CoV	Vero-E6, 72h	1462	35	
18β-glycyrrhetinic acid derivative 3	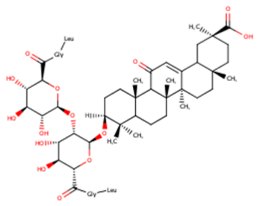	Pentacyclic triterpenes		SARS-CoV	Vero-E6, 72h	215	139	

Trivial name	Structure	Class of compounds	Plant	Viral strain	Host cells, incubation time	CC_{50} (μM)	EC_{50} (μM)	Ref. (DOI)
18β-glycyrrhetinic acid derivative 9	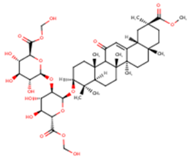	Pentacyclic triterpenes		SARS-CoV	Vero-E6, 72h	44	8	
3β-friedelanone	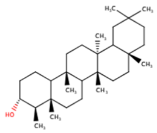	Pentacyclic triterpenes	*Euphorbia neriifolia*	HCoV-229E	MRC-5, 96h	-	132.4% uninfected cell survival	10.1177/1934578x1200701103
Aescin	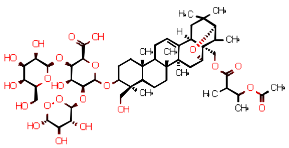	Pentacyclic triterpenes	*Aesculus hippocastanum*	SARS-CoV	Vero-E6, 72h	-	6	10.1073/pnas.0403596101
Betulonic acid	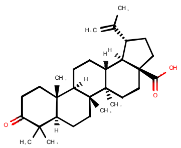	Pentacyclic triterpenes	*Juniperus formosana*	SARS-CoV	-	112	0.63	10.1021/jm070295s
Epitaraxerol	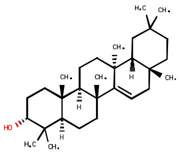	Pentacyclic triterpenes	*Euphorbia neriifolia*	HCoV-229E	-	-	111.0% uninfected cell survival	10.1177/1934578x1200701103
Friedelin	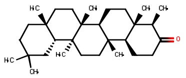	Pentacyclic triterpenes	*Euphorbia neriifolia*	HCoV-229E	-	-	109.0% uninfected cell survival	10.1177/1934578x1200701103

Trivial name	Structure	Class of compounds	Plant	Viral strain	Host cells, incubation time	CC_{50} (μM)	EC_{50} (μM)	Ref. (DOI)
Glycyrrhizin	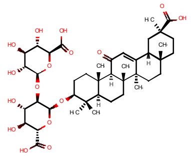	Pentacyclic triterpenes	*Glycyrrhiza sp.*	SARS-CoV	Vero-E6, 72h	>24000	365	10.1021/jm0493008
				SARS-CoV	Vero-E6, 72h	>20000	729.1	10.1016/S0140-6736(03)13615-X
Pristimerin	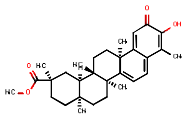	Pentacyclic triterpenes	-	HCoV-NL63	LLC-MK2, 72h	>20	1.63	10.1128/JVI.00023-19
				HCoV-OC43	BHK-21, 72h	>20	1.99	
				MERS-CoV	Vero-E6, 72h	>20	13.87	
Tylophorine	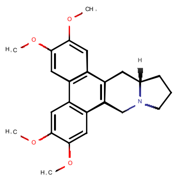	Phenanthraindolizidine alkaloids	*Tylophora indica*	SARS-CoV	Vero-E6, 72h	184.7	0.018	10.1016/j.antiviral.2010.08.009
Tylophorine N-oxide	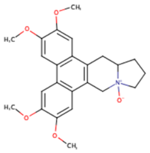	Phenanthraindolizidine alkaloids	*Tylophora indica*	SARS-CoV	Vero-E6, 72h	184.7	0.34	10.1016/j.antiviral.2010.08.009
Procyanidin A2	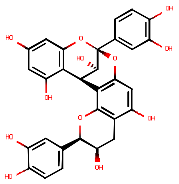	Proanthocyanidins	*Cinnamomum verum*	SARS-CoV	Vero-E6, 72h	1116.7	29.9	10.1016/j.antiviral.2009.02.001

Trivial name	Structure	Class of compounds	Plant	Viral strain	Host cells, incubation time	CC_{50} (µM)	EC_{50} (µM)	Ref. (DOI)
Procyanidin B1	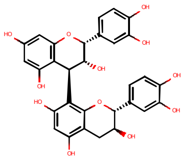	Proanthocyanidins	*Cinnamomum verum*	SARS-CoV	Vero-E6, 72h	648.2	41.3	10.1016/j.antiviral.2009.02.001
Bruceine A	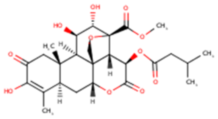	Quassinoids	*Brucea javanica*	SARS-CoV-2	Vero-E6, 72-96h	31.4	0.011	10.1038/s41392-020-00343-z
Chloroquine	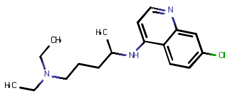	Quinoline alkaloids	*Nature derived*	HCoV-NL63	LLC-MK2, 72h	>20	4.89	10.1128/JVI.00023-19
				HCoV-OC43	BHK-21, 72h	>20	0.33	
				MERS-CoV	Vero-E6, 72h	>20	16.44	
				SARS-CoV	Vero	-	8.8	10.1016/j.bbrc.2004.08.085
Alantolactone	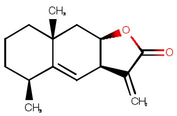	Sesquiterpene lactones	*Inula helenium*	SARS-CoV-2	Vero-E6, 72-96h	36.7	1.724	10.1038/s41392-020-00343-z
Dehydrocostus lactone	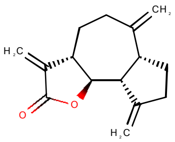	Sesquiterpene lactones	*Saussurea costus*	SARS-CoV-2	Vero-E6, 72-96h	36.2	2.322	10.1038/s41392-020-00343-z
Isoalantolactone	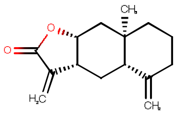	Sesquiterpene lactones	*Inula helenium*	SARS-CoV-2	Vero-E6, 72-96h	>40	1.483	10.1038/s41392-020-00343-z

Trivial name	Structure	Class of compounds	Plant	Viral strain	Host cells, incubation time	CC50 (μM)	EC50 (μM)	Ref. (DOI)
Cornuside	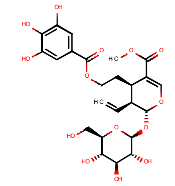	Secoiridoids	*Cornus officinalis*	SARS-CoV-2	Vero-E6, 72-96h	>40	5.262	10.1038/s41392-020-00343-z
Conessine	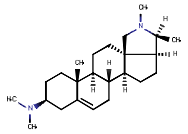	Steroidal alkaloids	-	HCoV-NL63	LLC-MK2, 72h	>20	10.75	10.1128/JVI.00023-19
				HCoV-OC43	BHK-21, 72h	>20	2.34	
				MERS-CoV	Vero-E6, 72h	>20	4.98	
Cinobufagin	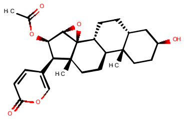	Steroids	*Toad Venom*	SARS-CoV-2	Vero-E6, 72-96h	>40	0.018	10.1038/s41392-020-00343-z
Veratridine	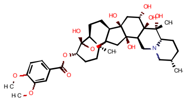	Steroids	-	SARS-CoV-2	Vero-E6, 72-96h	>100	2.376	10.1038/s41392-020-00343-z
Periplocoside	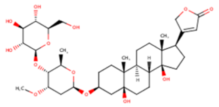	Steroids	*Toad Venom*	SARS-CoV-2	Vero-E6, 72-96h	>40	0.0657	10.1038/s41392-020-00343-z
Digoxin	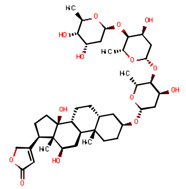	Steroids	*Digitalis*	SARS-CoV-2	Vero-E6, 72-96h	>40	0.1541	10.1038/s41392-020-00343-z

Trivial name	Structure	Class of compounds	Plant	Viral strain	Host cells, incubation time	CC_{50} (μM)	EC_{50} (μM)	Ref. (DOI)
Bufotalin	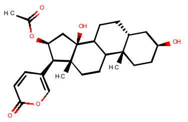	Steroids	*Toad Venom*	SARS-CoV-2	Vero-E6, 72-96h	>40	0.0259	10.1038/s41392-020-00343-z
Bufalin	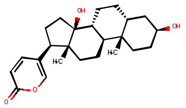	Steroids	*Toad Venom*	SARS-CoV-2	Vero-E6, 72-96h	>40	0.018	10.1038/s41392-020-00343-z
Cinnamtannin B1	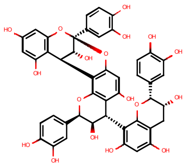	Tannins	*Cinnamomum verum*	SARS-CoV	Vero-E6, 72h	184.7	32.9	10.1016/j.antiviral.2009.02.001
Ginsenoside Rb1	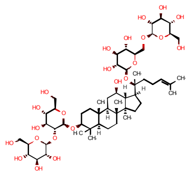	Triterpene saponins	*Panax sp.*	SARS-CoV	Vero-E6, 72h	-	100	10.1073/pnas.0403596101
Saikosaponin A	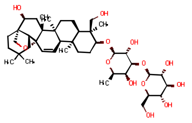	Triterpene saponins	*Bupleurum sp., Heteromorpha sp.*	HCoV-229E	MRC-5, 96h	228.1	8.6	10.1111/j.1440-1681.2006.04415.x

Trivial name	Structure	Class of compounds	Plant	Viral strain	Host cells, incubation time	CC_{50} (µM)	EC_{50} (µM)	Ref. (DOI)
Saikosaponin B2	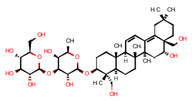	Triterpene saponins	*Bupleurum sp., Heteromorpha sp.*	HCoV-229E	MRC-5, 96h	383.3	1.7	10.1111/j.1440-1681.2006.04415.x
Saikosaponin C	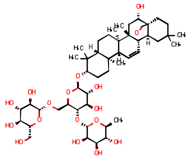	Triterpene saponins	*Bupleurum sp., Heteromorpha sp.*	HCoV-229E	MRC-5, 96h	121.5	19.9	10.1111/j.1440-1681.2006.04415.x
Saikosaponin D	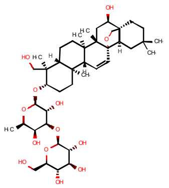	Triterpene saponins	*Bupleurum sp., Heteromorpha sp.*	HCoV-229E	MRC-5, 96h	176.2	13.2	10.1111/j.1440-1681.2006.04415.x
Momordinic	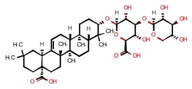	Triterpenes	*Bassia scoparia*	SARS-CoV-2	Vero-E6, 72-96h	>40	3.529	10.1038/s41392-020-00343-z
Brusatol	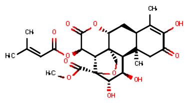	Triterpenes	*Brucea javanica*	SARS-CoV-2	Vero-E6, 72-96h	19	0.0492	10.1038/s41392-020-00343-z

**Trivial name**	**Structure**	**Class of compounds**	**Plant**	**Viral strain**	**Host cells, incubation time**	**CC_{50} (µM)**	**EC_{50} (µM)**	**Ref. (DOI)**
Roburic acid	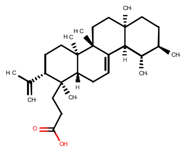	Triterpenes	*Gentiana macrophylla*	SARS-CoV-2	Vero-E6, 72-96h	>40	5.267	10.1038/s41392-020-00343-z
Rescinamine	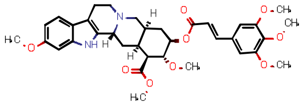	Vinca alkaloids	*Rauwolfia sp.*	SARS-CoV	Vero-E6, 72h	-	20	10.1073/pnas.0403596101
Reserpine	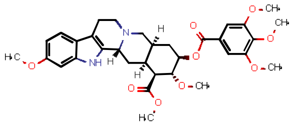	Vinca alkaloids	*Rauvolfia serpentina*	SARS-CoV	Vero-E6, 72h	No effect	3.4	10.1073/pnas.0403596101
Blancoxanthone	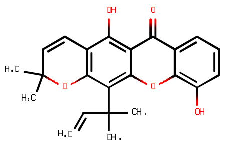	Xanthones	*Calophyllum blancoi*	HCoV-229E	MRC-5, 96h	-	7.9	10.1248/cpb.53.244

TABLE S 3 Inhibitory activity of extracts against the main protease of SARS-CoV, $3CL^{pro}$.

**Plant**	**Plant part / extract**	**Family**	**IC_{50} (µg/mL)**	**Reference**
Celastrus orbiculatus	whole algae/EtOH	Celastraceae	19.4	10.1517/13543776.2013.823159
Celastrus orbiculatus	whole algae/EtOAc	Celastraceae	17.8	
Celastrus orbiculatus	whole algae/water	Celastraceae	38.7	
Cibotium barometz	Roots/water	Cibotiaceae	>50	10.1016/S2225-4110(16)30055-4
Gentiana scabra	Roots/water	Gentianaceae	>50	
Cassia tora	Seeds/water	Fabaceae	>50	
Taxillus chinensis	Aerial parts/water	Loranthaceae	>50	
Dioscorea batatas	Tubers/water	Dioscoreaceae	44	
Isatis indigofera	Root/water	Brassicaceae	53.8	10.1016/j.antiviral.2005.07.002
Camellia sinensis	Black tea/water	Theaceae	70	Antiviral activity of phenolic polymers and cycloSal-pronucleotides against a SARS-associated coronavirus
Rheum palmatum	Whole plant/EtOH	Polygonaceae	38.09	Anti-SARS coronavirus 3C-like protease effects of Rheum palmatum L. extracts

TABLE S 4 Inhibition of HCoV enzymes from natural and nature-derived products.

**Name**		**Class**	**Plant name**	**Plant part**	**Family**	**Viral strain**	**Enzyme**	**IC_{50} (μM)**	**Reference**
β-sitosterol	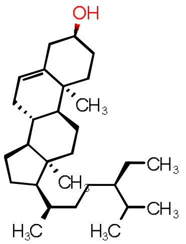	Steroids	*Isatis indigofera*	Root/water	Brassicaceae	SARS-CoV	3CLpro	115	10.1016/j.antiviral.2005.07.002
2-(3′,4′-Dihydroxyphenyl)-5,7-dihydroxy-3-b-D-arobinosyl-4H-chromen-4-one	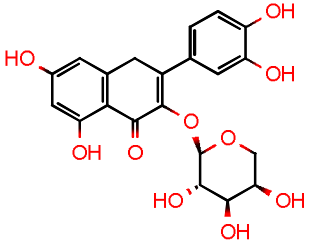	Flavonoids	Nature derived	-	-	SARS-CoV	3CLpro	31.62	10.1016/j.bmc.2006.09.014
2-(3′,4′-Dihydroxyphenyl)-5,7-dihydroxy-3-b-D-glucosyl-4H-chromen-4-one	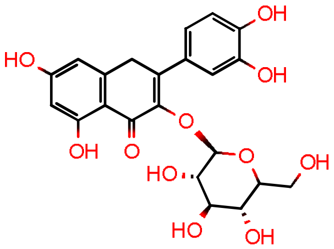	Flavonoids	Nature derived	-	-	SARS-CoV	3CLpro	48.85	10.1016/j.bmc.2006.09.014
2-(3′,4′-Dihydroxyphenyl)-5,7-dihydroxy-3-b-L-fucosyl-4H-chromen-4-one	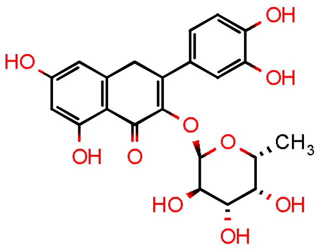	Flavonoids	Nature derived	-	-	SARS-CoV	3CLpro	24.14	10.1016/j.bmc.2006.09.014

Name		Class	Plant name	Plant part	Family	Viral strain	Enzyme	IC_{50} (μM)	Reference
2-(3′,4′-Dihydroxyphenyl)-5-hydroxy-3,7-di(b-D-galactosyl)-4H-chromen-4-one	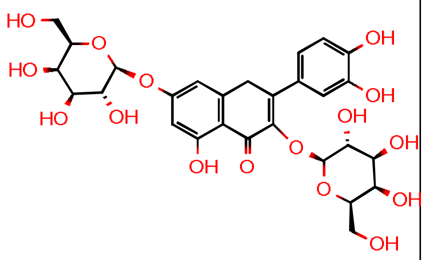	Flavonoids	Nature derived	-	-	SARS-CoV	3CLpro	61.46	10.1016/j.bmc.2006.09.014
2-Phloroeckol	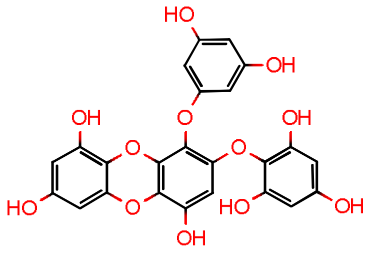	Tannins	*Ecklonia cava*	Whole algae/EtOH	Lessoniaceae	SARS-CoV	3CLpro	112.2	10.1016/j.bmc.2013.04.026
3′-(3-methylbut-2-enyl)-3′,4,7-trihydroxyflavane	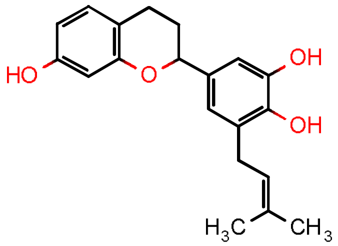	Flavonoids	*Broussonetia papyrifera*	Roots/EtOH	Moraceae	MERS-CoV	3CLpro	34.70	10.1080/14756366.2016.1265519
							PLpro	48.80	
						SARS-CoV	3CLpro	30.2	
							PLpro	35.8	
3′-O-methyldiplacol	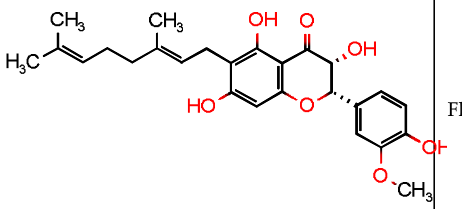	Flavonoids	*Paulownia tomentosa*	-	Scrophulariaceae	SARS-CoV	PLpro	9.5	10.1016/j.bmc.2013.03.027

Name		Class	Plant name	Plant part	Family	Viral strain	Enzyme	IC_{50} (μM)	Reference
3′-O-methyldiplacone	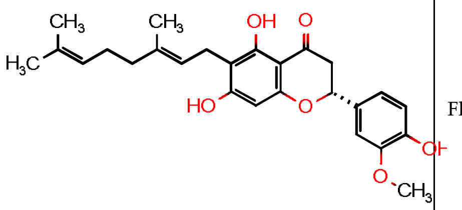	Flavonoids	*Paulownia tomentosa*	-	Scrophulariaceae	SARS-CoV	PLpro	13.2	
3-Isotheaflavin-3-gallate	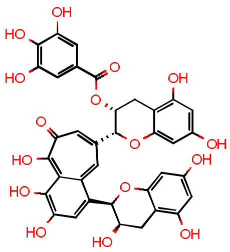	Theaflavins	*Camellia sinensis*	Black tea/water	Theaceae	SARS-CoV	3CLpro	9.8	M. Schmidtke, C. Meier, M. Schacke, B. Helbig, V. Makarov, H. F. Rabenau, J. Cinatl and P. Wutzler, Chemother. J., 2005, 14, 16–21.
4′-O-methylbavachalcone	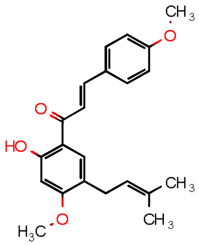	Chalcones	*Psoralea corylifolia*	Seeds/EtOH	Fabaceae	SARS-CoV	PLpro	10.1	10.3109/14756366.2012.753591
4′-O-methyldiplacol	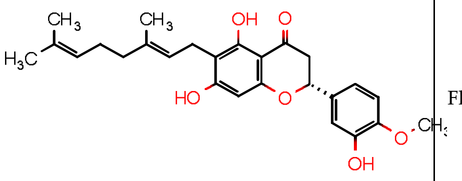	Flavonoids	*Paulownia tomentosa*	Fruits/MeOH	Scrophulariaceae	SARS-CoV	PLpro	9.2	10.1016/j.bmc.2013.03.027

Name		Class	Plant name	Plant part	Family	Viral strain	Enzyme	IC_{50} (µM)	Reference
4′-O-methyldiplacone	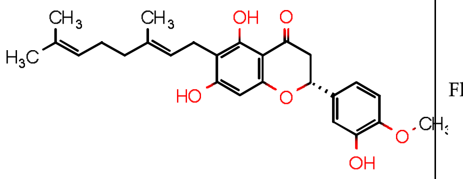	Flavonoids	*Paulownia tomentosa*	Fruits/MeOH	Scrophulariaceae	SARS-CoV	PLpro	12.7	
4-hydroxyisolonchocarpin	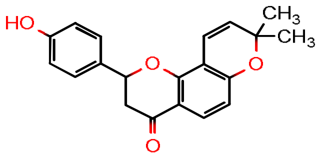	Flavonoids	*Paulownia tomentosa*	Fruits/MeOH	Scrophulariaceae	MERS-CoV	3CLpro<br>PLpro	193.70<br>171.60	10.1080/14756366.2016.1265519
		Flavonoids	*Paulownia tomentosa*	Fruits/MeOH	Scrophulariaceae	SARS-CoV	3CLpro<br>PLpro	202.7<br>35.4	10.1080/14756366.2016.1265519
6- geranyl-40,5,7-trihydroxy-3′,5′-dimethoxyflavanone	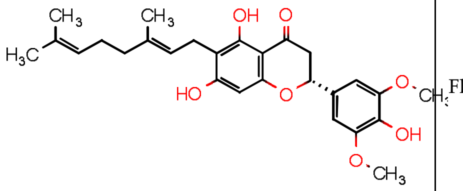	Flavonoids	*Paulownia tomentosa*	Fruits/MeOH	Scrophulariaceae	SARS-CoV	PLpro	13.9	10.1016/j.bmc.2013.03.027
7-Phloroeckol	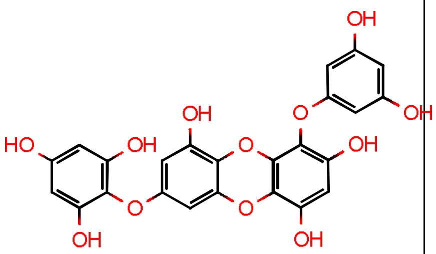	Tannins	*Ecklonia cava*	Whole algae/EtOH	Lessoniaceae	SARS-CoV	3CLpro	112	10.1016/j.bmc.2013.04.026
7-sulfonyl isatin derivative 7i	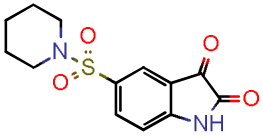	Indole alkaloids	Nature derived	-	-	SARS-CoV	3CLpro	4.45	10.1016/j.bmc.2013.11.028

Name		Class	Plant name	Plant part	Family	Viral strain	Enzyme	IC_{50} (μM)	Reference
7-sulfonyl isatin derivative 7k	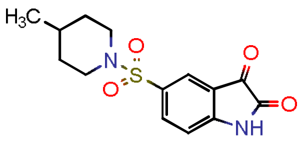	Indole alkaloids	Nature derived	-	-	SARS-CoV	3CLpro	1.18	10.1016/j.bmc.2013.11.028
7-sulfonyl isatin derivative 7l	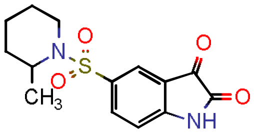	Indole alkaloids	Nature derived	-	-	SARS-CoV	3CLpro	2.25	10.1016/j.bmc.2013.11.028
7-sulfonyl isatin derivative 7m	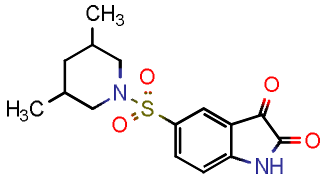	Indole alkaloids	Nature derived	-	-	SARS-CoV	3CLpro	4.3	10.1016/j.bmc.2013.11.028
7-sulfonyl, N-substituted isatin derivative 8k1	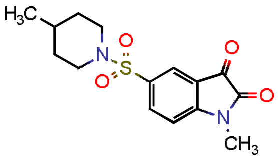	Indole alkaloids	Nature derived	-	-	SARS-CoV	3CLpro	1.04	10.1016/j.bmc.2013.11.028
7-sulfonyl, N-substituted isatin derivative 8k2	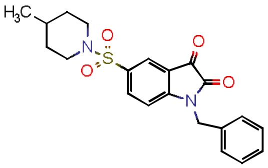	Indole alkaloids	Nature derived	-	-	SARS-CoV	3CLpro	1.69	10.1016/j.bmc.2013.11.028
aloe emodin	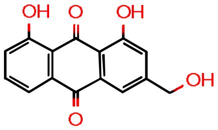	Anthraquinones	*Isatis indigofera*	Root/water	Brassicaceae	SARS-CoV	3CLpro	132	10.1016/j.antiviral.2005.07.002

Name		Class	Plant name	Plant part	Family	Viral strain	Enzyme	IC_{50} (μM)	Reference
Amentoflavone	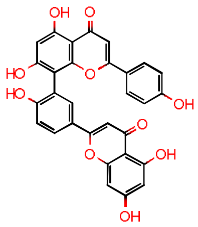	Flavonoids	*Torreya nucifera*	Leaves/EtOH	Taxaceae	SARS-CoV	3CLpro	8.3	10.1016/j.bmc.2010.09.035
Andrographolide	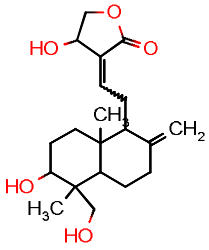	Labdane diterpenes	*Andrographis paniculata*	-	Acanthaceae	SARS-CoV-2	3CLpro	15.05	10.1016/j.bbrc.2020.08.086
Apigenin	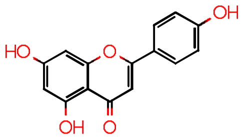	Flavonoids	*Torreya nucifera*	Leaves/EtOH	Taxaceae	SARS-CoV	3CLpro	280.8	10.1016/j.bmc.2010.09.035
Baicalein	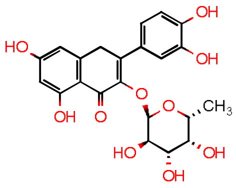	Flavonoids	*Scutellaria baicalensis*	-	Lamiaceae	SARS-CoV-2	3CLpro	0.94	10.1038/s41401-020-0483-6
Baicalin	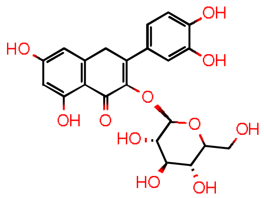	Flavonoids	*Scutellaria baicalensis*	-	Lamiaceae	SARS-CoV-2	3CLpro	6.41	10.1038/s41401-020-0483-6

**Name**		**Class**	**Plant name**	**Plant part**	**Family**	**Viral strain**	**Enzyme**	**IC_{50} (μM)**	**Reference**
Bavachinin	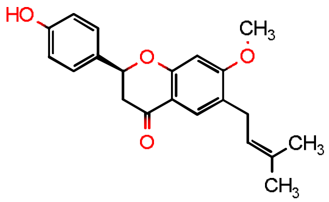	Isoflavones	*Psoralea corylifolia*	Seeds/EtOH	Fabaceae	SARS-CoV	PLpro	38.4	10.3109/14756366.2012.753591
Betulinic acid	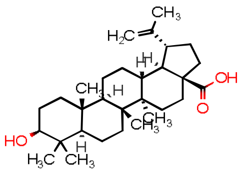	pentacyclic triterpenes	*Juniperus formosana*	-	-	SARS-CoV	3CLpro	10	10.1021/jm070295s
Betulonic acid	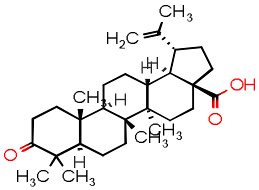	pentacyclic triterpenes	-	-	-	SARS-CoV	3CLpro	>100	10.1021/jm070295s
Bilobetin	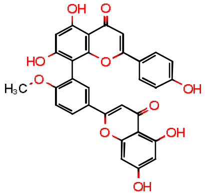	Flavonoids	*Torreya nucifera*	Leaves/EtOH	Taxaceae	SARS-CoV	3CLpro	72.3	10.1016/j.bmc.2010.09.035
Broussochalcone A	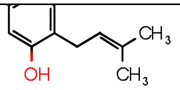	Chalcones	*Broussonetia papyrifera*	Roots/EtOH	Moraceae	MERS-CoV<br>SARS-CoV	3CLpro<br>PLpro<br>3CLpro	36.20<br>42.10<br>88.1	10.1080/14756366.2016.1265519

Name		Class	Plant name	Plant part	Family	Viral strain	Enzyme	IC_{50} (μM)	Reference
							PLpro	9.2	
Broussochalcone B	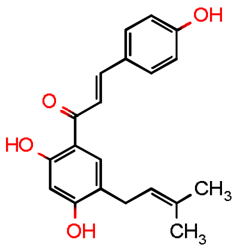	Chalcones	*Broussonetia papyrifera*	Roots/EtOH	Moraceae	MERS-CoV	3CLpro	27.90	
							PLpro	112.90	
						SARS-CoV	3CLpro	57.8	10.1080/14756366.2016.1265519
							PLpro	11.6	
Broussoflavan A	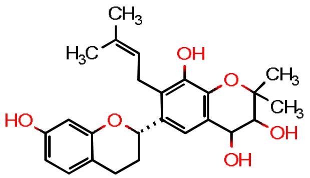	Flavonoids	*Broussonetia papyrifera*	Roots/EtOH	Moraceae	MERS-CoV	3CLpro	125.70	
							PLpro	49.10	
						SARS-CoV	3CLpro	92.4	
							PLpro	30.4	
Celastrol	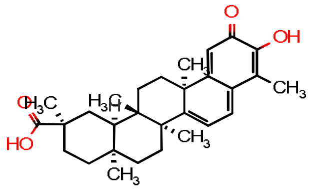	Pentacyclic triterpenes	*Tripterygium regelii*	Roots/MeOH	Celastraceae	SARS-CoV	3CLpro	10.3	10.1016/j.bmcl.2010.01.152
Chalcone derivative 1	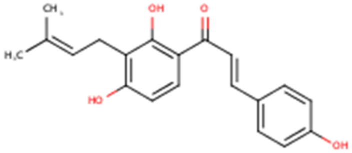	Chalcones	*Angelica keiskei*	Leaves/EtOH	Apiaceae	SARS-CoV	3CLpro	39.4	10.3109/14756366.2014.1003215
							PLpro	13	

Name		Class	Plant name	Plant part	Family	Viral strain	Enzyme	IC_{50} (μM)	Reference
Chalcone derivative 2	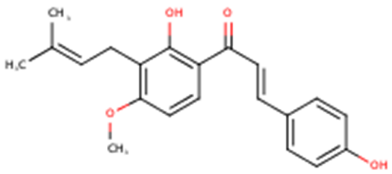	Chalcones	*Angelica keiskei*	Leaves/EtOH	Apiaceae	SARS-CoV	3CLpro<br>PLpro	81.4<br>26	10.3109/14756366.2014.1003215
Chalcone derivative 3	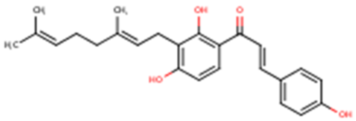	Chalcones	*Angelica keiskei*	Leaves/EtOH	Apiaceae	SARS-CoV	3CLpro<br>PLpro	38.4<br>11.7	10.3109/14756366.2014.1003215
Chalcone derivative 4	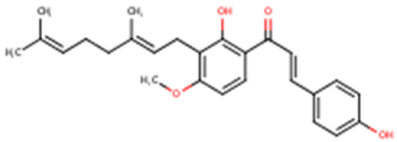	Chalcones	*Angelica keiskei*	Leaves/EtOH	Apiaceae	SARS-CoV	3CLpro<br>PLpro	34.1<br>5.6	10.3109/14756366.2014.1003215
Chalcone derivative 5	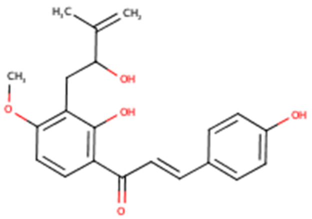	Chalcones	*Angelica keiskei*	Leaves/EtOH	Apiaceae	SARS-CoV	3CLpro<br>PLpro	26.6<br>19.3	10.3109/14756366.2014.1003215
Chalcone derivative 6	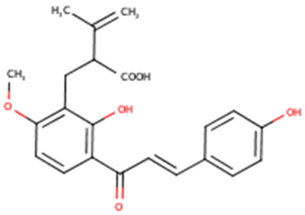	Chalcones	*Angelica keiskei*	Leaves/EtOH	Apiaceae	SARS-CoV	3CLpro<br>PLpro	11.4<br>1.2	10.3109/14756366.2014.1003215

Name		Class	Plant name	Plant part	Family	Viral strain	Enzyme	IC_{50} (μM)	Reference
Chalcone derivative 7	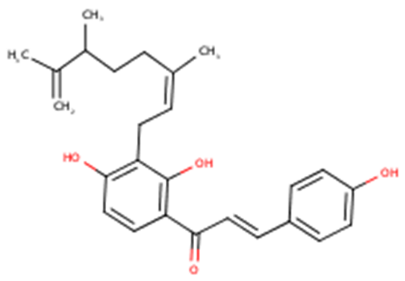	Chalcones	*Angelica keiskei*	Leaves/EtOH	Apiaceae	SARS-CoV	3CLpro<br>PLpro	22.2<br>11.7	10.3109/14756366.2014.1003215
Chalcone derivative 8	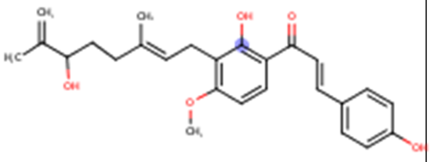	Chalcones	*Angelica keiskei*	Leaves/EtOH	Apiaceae	SARS-CoV	3CLpro<br>PLpro	189.8<br>46.4	10.3109/14756366.2014.1003215
Chalcone derivative 9	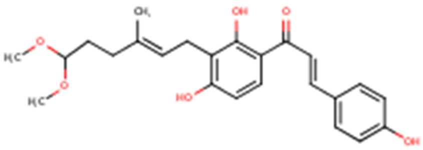	Chalcones	*Angelica keiskei*	Leaves/EtOH	Apiaceae	SARS-CoV	3CLpro<br>PLpro	44.1<br>21.1	10.3109/14756366.2014.1003215
Cinnamic acid amide 1	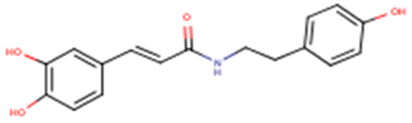	Cinnamates	*Tribulus terrestris*	Fruits/MeOH	Zygophyllaceae	SARS-CoV	PLpro	44.4	10.1248/bpb.b14-00026
Cinnamic acid amide 2	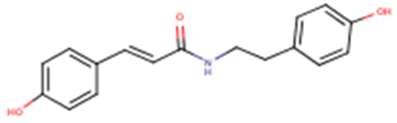	Cinnamates	*Tribulus terrestris*	Fruits/MeOH	Zygophyllaceae	SARS-CoV	PLpro	38.8	10.1248/bpb.b14-00026
Cinnamic acid amide 3	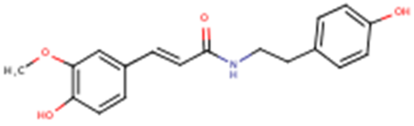	Cinnamates	*Tribulus terrestris*	Fruits/MeOH	Zygophyllaceae	SARS-CoV	PLpro	70.1	10.1248/bpb.b14-00026

Name		Class	Plant name	Plant part	Family	Viral strain	Enzyme	IC_{50} (μM)	Reference
Cinnamic acid amide 4	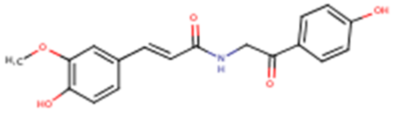	Cinnamates	*Tribulus terrestris*	Fruits/MeOH	Zygophyllaceae	SARS-CoV	PLpro	21.5	10.1248/bpb.b14-00026
Cinnamic acid amide 5	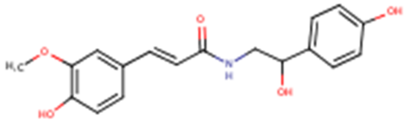	Cinnamates	*Tribulus terrestris*	Fruits/MeOH	Zygophyllaceae	SARS-CoV	PLpro	26.6	10.1248/bpb.b14-00026
Cinnamic acid amide 6	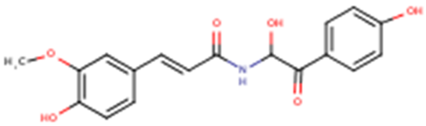	Cinnamates	*Tribulus terrestris*	Fruits/MeOH	Zygophyllaceae	SARS-CoV	PLpro	15.8	10.1248/bpb.b14-00026
Cinnamic acid amide 7	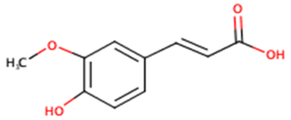	Cinnamates	*Tribulus terrestris*	Fruits/MeOH	Zygophyllaceae	SARS-CoV	PLpro	200	10.1248/bpb.b14-00026
Corylifol A	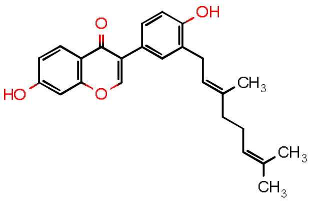	Isoflavones	*Psoralea corylifolia*	Seeds/EtOH	Fabaceae	SARS-CoV	PLpro	32.3	10.3109/14756366.2012.753591
Cryptotanshinone	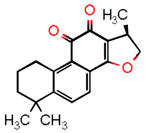	Diterpenes - phenanthrenes	*Salvia miltiorrhiza*	Roots/EtOH	Lamiaceae	SARS-CoV	3CLpro	226.7	10.1016/j.bmc.2012.07.038.
							PLpro	0.8	
Curcumin	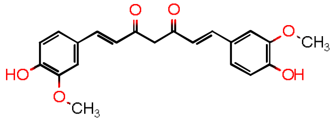	Diarylheptanoids	-	-	-	SARS-CoV	3CLpro	40	10.1021/jm070295s
						SARS-CoV	PLpro	5.7	10.1248/bpb.b12-00623

Name		Class	Plant name	Plant part	Family	Viral strain	Enzyme	IC_{50} (μM)	Reference
Daidzein	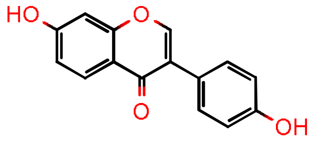	Isoflavones	*Isatis indigofera*	Root/water	Brassicaceae	SARS-CoV	3CLpro	105	10.1016/j.antiviral.2005.07.002
Dieckol	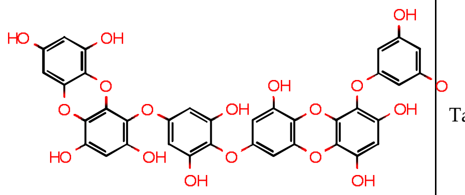	Tannins	*Ecklonia cava*	Whole algae/EtOH	Lessoniaceae	SARS-CoV	3CLpro	68.1	10.1016/j.bmc.2013.04.026
Dihydrotanshinone I	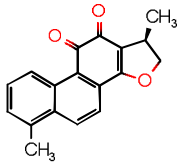	Diterpenes - phenanthrenes	*Salvia miltiorrhiza*	Roots/EtOH	Lamiaceae	SARS-CoV	3CLpro<br>PLpro	14.4<br>4.9	10.1016/j.bmc.2012.07.038.
Dioxinodehydroeckol	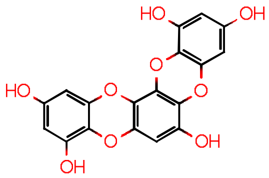	Tannins	*Ecklonia cava*	Whole algae/EtOH	Lessoniaceae	SARS-CoV	3CLpro	146.5	10.1016/j.bmc.2013.04.026
Diplacone	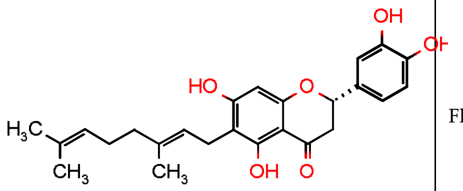	Flavonoids	*Paulownia tomentosa*	Fruits/MeOH	Scrophulariaceae	SARS-CoV	PLpro	10.4	10.1016/j.bmc.2013.03.027

Name		Class	Plant name	Plant part	Family	Viral strain	Enzyme	IC_{50} (μM)	Reference
Fucodiphloroethol G	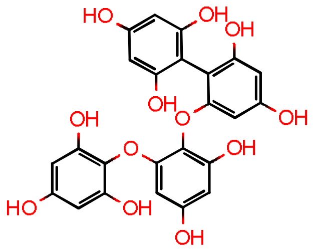	Tannins	*Ecklonia cava*	Whole algae/EtOH	Lessoniaceae	SARS-CoV	3CLpro	177.1	10.1016/j.bmc.2013.04.026
Ginkgetin	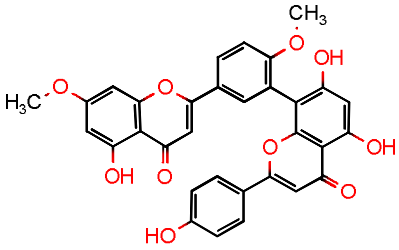	Flavonoids	*Torreya nucifera*	Leaves/EtOH	Taxaceae	SARS-CoV	3CLpro	32	10.1016/j.bmc.2010.09.035
Herbacetin	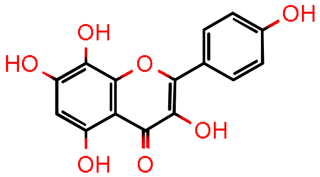	Flavonoids	-	-	-	SARS-CoV	3CLpro	33.17	10.1080/14756366.2019.1690480
Hesperetin	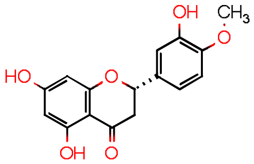	Flavonoids	*Isatis indigofera*	Root/water	Brassicaceae	SARS-CoV	3CLpro	60	10.1016/j.antiviral.2005.07.002
Hinokinin	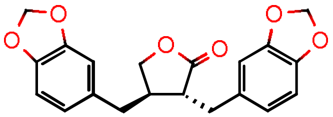	Lignans	*Chamaecyparis obtusa*	Heartwood/EtOAc	Cupressaceae	SARS-CoV	3CLpro	>100	10.1021/jm070295s

Name		Class	Plant name	Plant part	Family	Viral strain	Enzyme	IC_{50} (μM)	Reference
Hirsutanonol	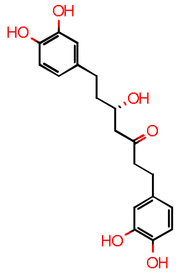	Diarylheptanoids	*Alnus japonica*	Bark/EtOH	Betulaceae	SARS-CoV	3CLpro	105.6	10.1248/bpb.b12-00623
							PLpro	7.8	
Hirsutenone	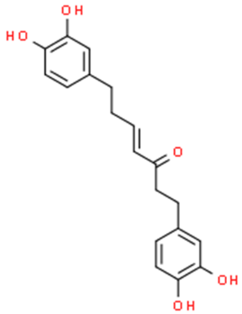	Diarylheptanoids	*Alnus japonica*	Bark/EtOH	Betulaceae	SARS-CoV	3CLpro	36.2	10.1248/bpb.b12-00623
							PLpro	4.1	
Hyperoside	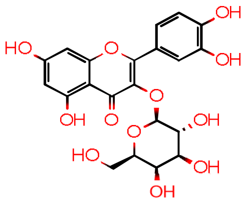	Flavonoids	*Broussonetia papyrifera*	Roots/EtOH	Moraceae	MERS-CoV	3CLpro	68.00	10.1080/14756366.2016.1265519
							PLpro	129.40	
			-	-	-	SARS-CoV	3CLpro	42.79	10.1016/j.bmc.2006.09.014
			Broussonetia papyrifera	Roots/EtOH	Moraceae	SARS-CoV	3CLpro	128.8	10.1080/14756366.2016.1265519
							PLpro	51.9	
Iguesterin	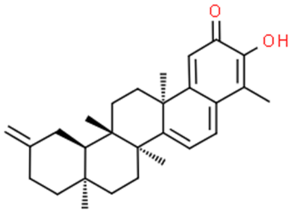	Pentacyclic triterpenes	*Tripterygium regelii*	Roots/MeOH	Celastraceae	SARS-CoV	3CLpro	2.6	10.1016/j.bmcl.2010.01.152

Name		Class	Plant name	Plant part	Family	Viral strain	Enzyme	IC_{50} (μM)	Reference
Indican	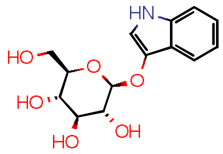	Indole alkaloids	*Isatis indigofera*	Root/water	Brassicaceae	SARS-CoV	3CLpro	112	10.1016/j.antiviral.2005.07.002
Indigo	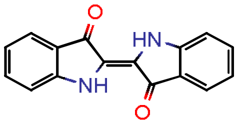	Indole alkaloids	*Isatis indigofera*	Root/water	Brassicaceae	SARS-CoV	3CLpro	300	10.1016/j.antiviral.2005.07.002
Indirubin	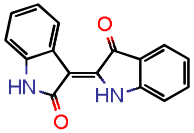	Indole alkaloids	*Isatis indigofera*	Root/water	Brassicaceae	SARS-CoV	3CLpro	293	10.1016/j.antiviral.2005.07.002
Isatin derivative 4b	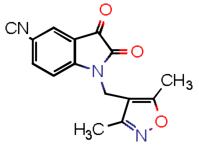	Indole alkaloids	Nature derived	-	-	SARS-CoV	3CLpro	7.2	10.1016/j.bmcl.2005.04.027
Isatin derivative 4c	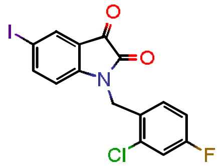	Indole alkaloids	Nature derived	-	-	SARS-CoV	3CLpro	9.4	10.1016/j.bmcl.2005.04.027
Isatin derivative 4d	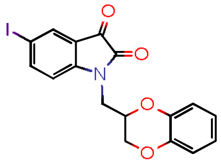	Indole alkaloids	Nature derived	-	-	SARS-CoV	3CLpro	13.5	10.1016/j.bmcl.2005.04.027
Isatin derivative 4h	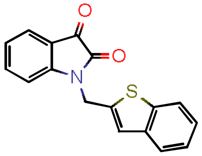	Indole alkaloids	Nature derived	-	-	SARS-CoV	3CLpro	13.11	10.1016/j.bmcl.2005.04.027

Name		Class	Plant name	Plant part	Family	Viral strain	Enzyme	IC_{50} (μM)	Reference
Isatin derivative 4i	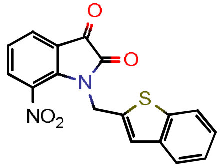	Indole alkaloids	Nature derived	-	-	SARS-CoV	3CLpro	2	10.1016/j.bmcl.2005.04.027
Isatin derivative 4k	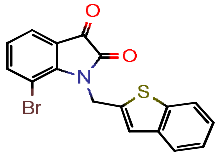	Indole alkaloids	Nature derived	-	-	SARS-CoV	3CLpro	0.98	10.1016/j.bmcl.2005.04.027
Isatin derivative 4n	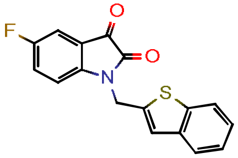	Indole alkaloids	Nature derived	-	-	SARS-CoV	3CLpro	4.82	10.1016/j.bmcl.2005.04.027
Isatin derivative 4o	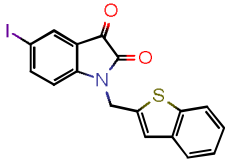	Indole alkaloids	Nature derived	-	-	SARS-CoV	3CLpro	0.95	10.1016/j.bmcl.2005.04.027
Isatin derivative 4p	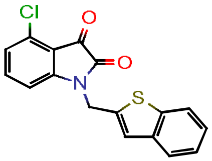	Indole alkaloids	Nature derived	-	-	SARS-CoV	3CLpro	11.2	10.1016/j.bmcl.2005.04.027
Isatin derivative 4r	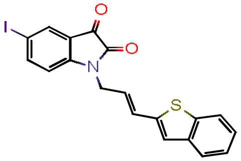	Indole alkaloids	Nature derived	-	-	SARS-CoV	3CLpro	23.5	10.1016/j.bmcl.2005.04.027

Name		Class	Plant name	Plant part	Family	Viral strain	Enzyme	IC50 (µM)	Reference
Isobavachalcone	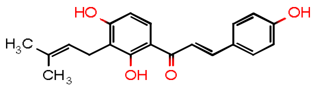	Chalcones	*Psoralea corylifolia*	Seeds/EtOH	Fabaceae	SARS-CoV	PLpro	7.3	10.3109/14756366.2012.753591
Isoliquiritigenin	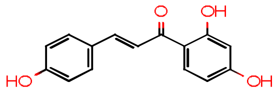	Chalcones	*Broussonetia papyrifera*	Roots/EtOH	Moraceae	MERS-CoV<br>SARS-CoV	3CLpro<br>PLpro<br>3CLpro<br>PLpro	33.90<br>82.20<br>61.9<br>24.6	10.1080/14756366.2016.1265519
Kaempferol	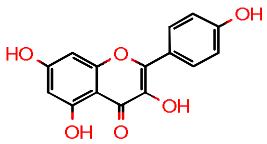	Flavonoids	*Broussonetia papyrifera*	Roots/EtOH	Moraceae	MERS-CoV<br>SARS-CoV	3CLpro<br>PLpro<br>3CLpro<br>PLpro	35.30<br>206.60<br>116.3<br>16.3	10.1080/14756366.2016.1265519
Kazinol A	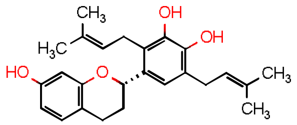	Diarylpropanoids	*Broussonetia papyrifera*	Roots/EtOH	Moraceae	MERS-CoV<br>SARS-CoV	3CLpro<br>PLpro<br>3CLpro<br>PLpro	-<br>88.50<br>84.8<br>66.2	10.1080/14756366.2016.1265519
Kazinol B	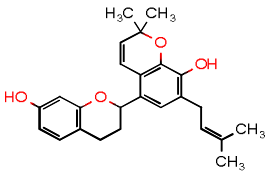	Diarylpropanoids	*Broussonetia papyrifera*	Roots/EtOH	Moraceae	MERS-CoV<br>SARS-CoV	3CLpro<br>PLpro<br>3CLpro<br>PLpro	-<br>94.90<br>233.3<br>31.4	10.1080/14756366.2016.1265519
Kazinol F	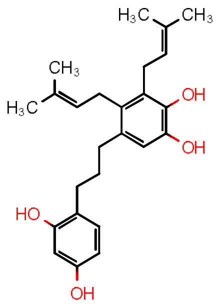	Diarylpropanoids	*Broussonetia papyrifera*	Roots/EtOH	Moraceae	MERS-CoV<br>SARS-CoV	3CLpro<br>PLpro<br>3CLpro<br>PLpro	135.00<br>39.50<br>43.3<br>27.8	10.1080/14756366.2016.1265519

Name		Class	Plant name	Plant part	Family	Viral strain	Enzyme	IC_{50} (μM)	Reference
Kazinol J	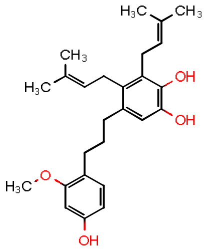	Diarylpropanoids	*Broussonetia papyrifera*	Roots/EtOH	Moraceae	MERS-CoV	3CLpro	109.20	10.1080/14756366.2016.1265519
							PLpro	55.00	
						SARS-CoV	3CLpro	64.2	
							PLpro	15.2	
Luteolin	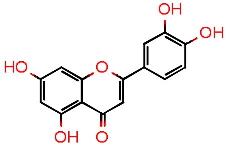	Flavonoids	*Torreya nucifera*	Leaves/EtOH	Taxaceae	SARS-CoV	3CLpro	20	10.1016/j.bmc.2010.09.035
Methyl tanshinonate	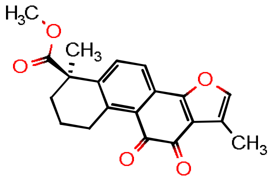	Diterpenes - phenanthrenes	*Salvia miltiorrhiza*	Roots/EtOH	Lamiaceae	SARS-CoV	3CLpro	21.1	10.1016/j.bmc.2012.07.038.
							PLpro	9.2	
Mimulone	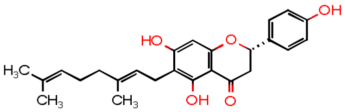	Flavonoids	*Paulownia tomentosa*	Fruits/MeOH	Scrophulariaceae	SARS-CoV	PLpro	14.4	10.1016/j.bmc.2013.03.027
Myricetin	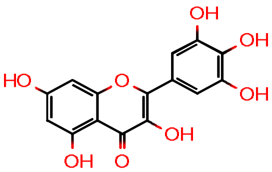	Flavonoids	-	-	-	SARS-CoV	nsP13	2.71	10.1016/j.bmcl.2012.04.081

Name		Class	Plant name	Plant part	Family	Viral strain	Enzyme	IC_{50} (μM)	Reference
Neobavaisoflavone	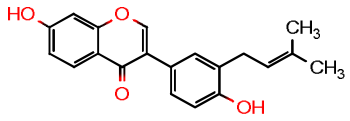	Isoflavones	*Psoralea corylifolia*	Seeds/EtOH	Fabaceae	SARS-CoV	PLpro	18.3	10.3109/14756366.2012.753591
Oregonin	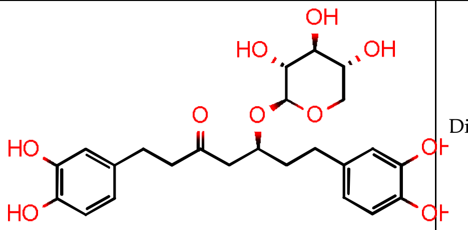	Diarylheptanoids	*Alnus japonica*	Bark/EtOH	Betulaceae	SARS-CoV	3CLpro	129.5	10.1248/bpb.b12-00623
							PLpro	20.1	
Papyriflavonol A	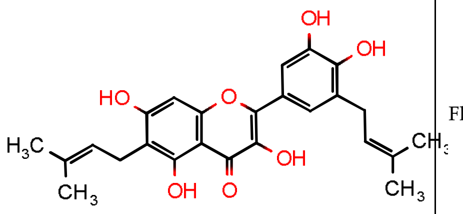	Flavonoids	*Broussonetia papyrifera*	Roots/EtOH	Moraceae	MERS-CoV	3CLpro	64.50	10.1080/14756366.2016.1265519
							PLpro	112.50	
						SARS-CoV	3CLpro	103.6	
							PLpro	3.7	
Pectolinarin	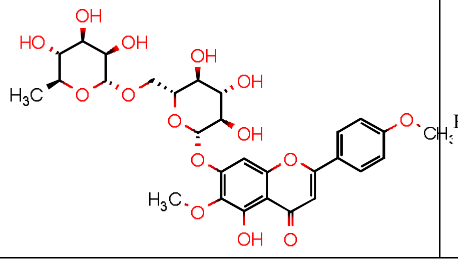	Flavonoids				SARS-CoV	3CLpro	37.78	10.1080/14756366.2019.1690480

Name		Class	Plant name	Plant part	Family	Viral strain	Enzyme	IC50 (μM)	Reference
Phlorofucofuroeckol A	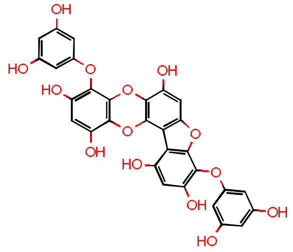	Tannins	*Ecklonia cava*	Whole algae/EtOH	Lessoniaceae	SARS-CoV	3CLpro	174.6	10.1016/j.bmc.2013.04.026
Platyphyllenone	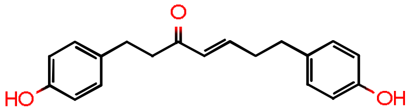	Diarylheptanoids	*Alnus japonica*	Bark/EtOH	Betulaceae	SARS-CoV	3CLpro<br>PLpro	>200<br>>200	10.1248/bpb.b12-00623
Platyphyllone	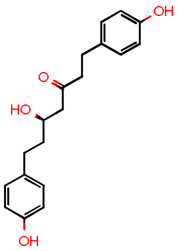	Diarylheptanoids	*Alnus japonica*	Bark/EtOH	Betulaceae	SARS-CoV	3CLpro<br>PLpro	>200<br>>200	10.1248/bpb.b12-00623
Platyphyllonol-5-xylopyranoside	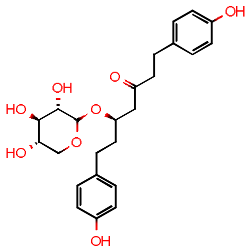	Diarylheptanoids	*Alnus japonica*	Bark/EtOH	Betulaceae	SARS-CoV	3CLpro<br>PLpro	>200<br>>200	10.1248/bpb.b12-00623

Name		Class	Plant name	Plant part	Family	Viral strain	Enzyme	IC_{50} (μM)	Reference
Pristimerin	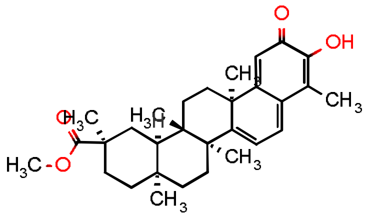	Pentacyclic triterpenes	*Tripterygium regelii*	Roots/MeOH	Celastraceae	SARS-CoV	3CLpro	5.5	10.1016/j.bmcl.2010.01.152
Psoralidin	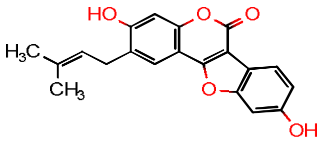	Coumarins	*Psoralea corylifolia*	Seeds/EtOH	Fabaceae	SARS-CoV	PLpro	4.2	10.3109/14756366.2012.753591
Quercetin	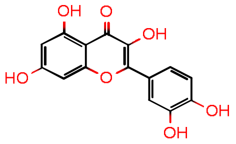	Flavonoids	*Broussonetia papyrifera*	Roots/EtOH	Moraceae	MERS-CoV	3CLpro	34.80	10.1080/14756366.2016.1265519
							PLpro	n.d.	
			Torreya nucifera	Leaves/EtOH	Taxaceae	SARS-CoV	3CLpro	23.8	10.1016/j.bmc.2010.09.035
			Broussonetia papyrifera	Roots/EtOH	Moraceae	SARS-CoV	3CLpro	52.7	10.1080/14756366.2016.1265519
							PLpro	8.6	
Rhoifolin	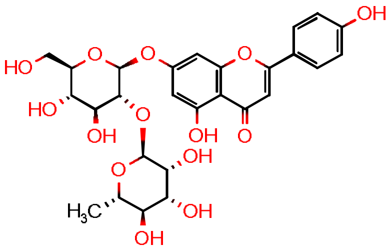	Flavonoids	-	-	-	SARS-CoV	3CLpro	27.45	10.1080/14756366.2019.1690480
Rosmariquinone	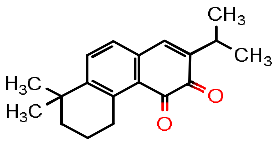	Diterpenes - phenanthrenes	*Salvia miltiorrhiza*	Roots/EtOH	Lamiaceae	SARS-CoV	3CLpro	21.1	10.1016/j.bmc.2012.07.038.
							PLpro	30	

Name		Class	Plant name	Plant part	Family	Viral strain	Enzyme	IC_{50} (μM)	Reference
Rubranol	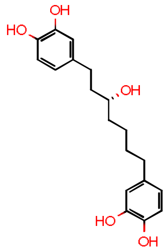	Diarylheptanoids	*Alnus japonica*	Bark/EtOH	Betulaceae	SARS-CoV	3CLpro<br>PLpro	144.6<br>12.3	10.1248/bpb.b12-00623
Rubranoside A	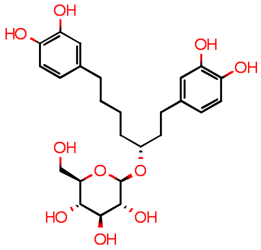	Diarylheptanoids	*Alnus japonica*	Bark/EtOH	Betulaceae	SARS-CoV	3CLpro<br>PLpro	102.1<br>9.1	10.1248/bpb.b12-00623
Rubranoside B	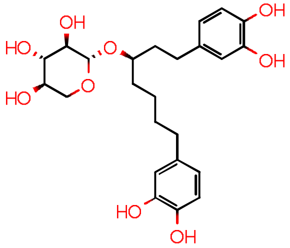	Diarylheptanoids	*Alnus japonica*	Bark/EtOH	Betulaceae	SARS-CoV	3CLpro<br>PLpro	105.3<br>8	10.1248/bpb.b12-00623
Savinin	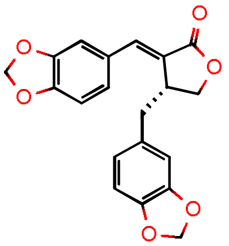	Lignans	*Chamaecyparis obtusa*	Heartwood/ EtOAc	Cupressaceae	SARS-CoV	3CLpro	25	10.1021/jm070295s

Name		Class	Plant name	Plant part	Family	Viral strain	Enzyme	IC_{50} (μM)	Reference
Sciadopitysin	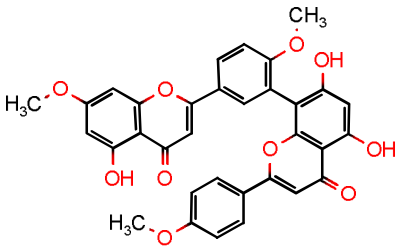	Flavonoids	*Torreya nucifera*	Leaves/EtOH	Taxaceae	SARS-CoV	3CLpro	38.4	10.1016/j.bmc.2010.09.035
Scutellarein	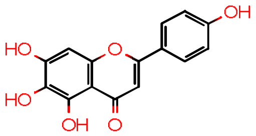	Flavonoids	-	-		SARS-CoV	nsP13	0.86	10.1016/j.bmcl.2012.04.081
Sinigrin	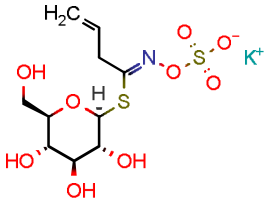	Glucosinolates	*Isatis indigofera*	Root/water	Brassicaceae	SARS-CoV	3CLpro	121	10.1016/j.antiviral.2005.07.002
Tannic acid	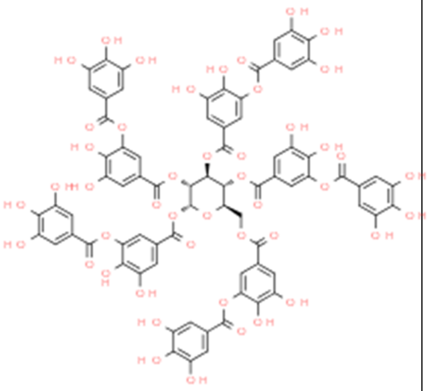	Tanins				SARS-CoV-2	3CLpro	2.1	10.1371/journal.pone.0240079

**Name**		**Class**	**Plant name**	**Plant part**	**Family**	**Viral strain**	**Enzyme**	**IC$_{50}$ (μM)**	**Reference**
Tanshinone I	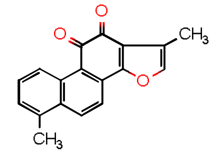	Diterpenes - phenanthrenes	*Salvia miltiorrhiza*	Roots/EtOH	Lamiaceae	SARS-CoV	3CLpro<br>PLpro	38.7<br>8.8	10.1016/j.bmc.2012.07.038.
Tanshinone IIA	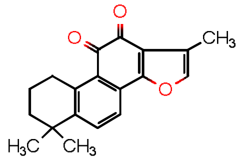	Diterpenes - phenanthrenes	*Salvia miltiorrhiza*	Roots/EtOH	Lamiaceae	SARS-CoV	3CLpro<br>PLpro	89.1<br>1.6	10.1016/j.bmc.2012.07.038.
Tanshinone IIB	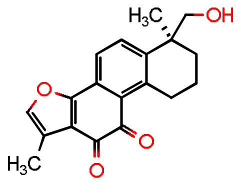	Diterpenes - phenanthrenes	*Salvia miltiorrhiza*	Roots/EtOH	Lamiaceae	SARS-CoV	3CLpro<br>PLpro	24.8<br>10.7	10.1016/j.bmc.2012.07.038.
Theaflavin-3,3'-digallate	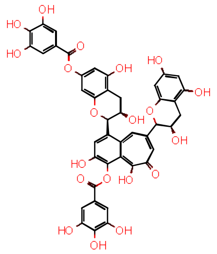	Theaflavins	*Camellia sinensis*	Black tea/water	Theaceae	SARS-CoV	3CLpro	10.9	M. Schmidtke, C. Meier, M. Schacke, B. Helbig, V. Makarov, H. F. Rabenau, J. Cinatl and P. Wutzler, Chemother. J., 2005, 14, 16–21.
Tingenone	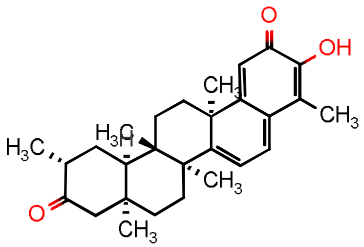	pentacyclic triterpenes	*Tripterygium regelii*	Roots/MeOH	Celastraceae	SARS-CoV	3CLpro	10	10.1016/j.bmcl.2010.01.152

Name		Class	Plant name	Plant part	Family	Viral strain	Enzyme	IC_{50} (μM)	Reference
Tomentin A	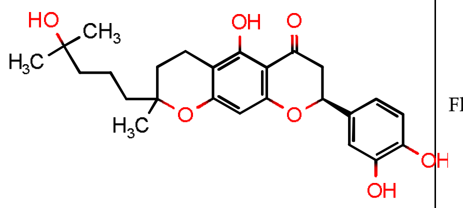	Flavonoids	*Paulownia tomentosa*	Fruits/MeOH	Scrophulariaceae	SARS-CoV	PLpro	6	10.1016/j.bmc.2013.03.027
Tomentin B	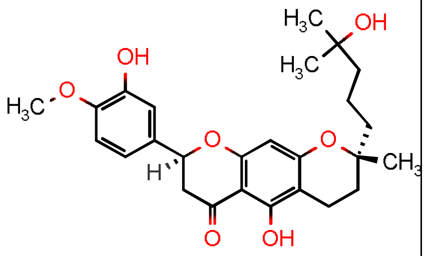	Flavonoids	*Paulownia tomentosa*	Fruits/MeOH	Scrophulariaceae	SARS-CoV	PLpro	6	10.1016/j.bmc.2013.03.027
Tomentin C	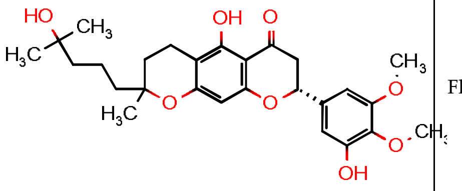	Flavonoids	*Paulownia tomentosa*	Fruits/MeOH	Scrophulariaceae	SARS-CoV	PLpro	12	10.1016/j.bmc.2013.03.027
Tomentin D	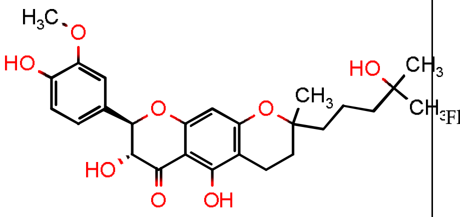	Flavonoids	*Paulownia tomentosa*	Fruits/MeOH	Scrophulariaceae	SARS-CoV	PLpro	13	10.1016/j.bmc.2013.03.027

Name		Class	Plant name	Plant part	Family	Viral strain	Enzyme	IC_{50} (μM)	Reference
Tomentin E	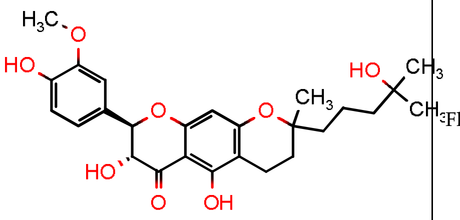	Flavonoids	*Paulownia tomentosa*	Fruits/MeOH	Scrophulariaceae	SARS-CoV	PLpro	5	10.1016/j.bmc.2013.03.027
